# Effects of cropping patterns and nitrogen application rates on soil microbial community characteristics in goji berry root zones

**DOI:** 10.3389/fpls.2026.1793632

**Published:** 2026-04-02

**Authors:** Chongqin Luo, Fusen Yang, Yuhong Pu, Yanxia Kang, Yanlin Ma, Guangping Qi, Chungang Jing, Huile Lv, Haiyan Li, Yuanbo Jiang, Mingzhu Wang

**Affiliations:** 1 College of Water Conservancy and Hydropower Engineering, Gansu Agricultural University, Lanzhou, China; 2 Qingyang Hydrological and Water Resources Survey Center, Gansu, Qingyang, China

**Keywords:** arid saline–alkaline region, goji berry, intercropping pattern, nitrogen application rate, root zone microorganisms

## Abstract

**Abstract:**

Goji berry planting in arid saline-alkali areas faces the challenges of soil micro-ecological imbalance and excessive application of nitrogen fertilizer.

**Objectives:**

To clarify the interactive effects of different planting patterns and nitrogen application levels on the structure, diversity and function of soil microbial communities in the root zone of goji berry in arid saline-alkali areas, which is of great significance for optimizing the planting system of goji berry in this area and reducing nitrogen application rates and improving nitrogen use efficiency.

**Methods:**

Based on field experiments, this study set up two planting patterns: goji berry monoculture and goji–alfalfa intercropping, four nitrogen application gradients: 0 kg·hm^-^² (N0), 150 kg·hm^-^² (N1), 300 kg·hm^-^² (N2), and 450 kg·hm^-^² (N3). The culturable microbial counts, community structure, diversity and functional genes of bacteria and fungi were analyzed by dilution coating plate method, high-throughput sequencing and functional prediction. Results: Compared with monoculture, the abundances of culturable soil bacteria and actinomycetes were significantly increased under the goji–alfalfa intercropping pattern, while the abundance of culturable fungi and the relative abundance of potential pathogens were inhibited, and the bacterial community structure was optimized. For example, the relative abundance of *Proteobacteria* decreased, and the proportion of *Gemmatimonadota*, *Actinomycetota* and *Thermomicrobiota*, increased. Diversity analysis showed that N1 treatment was beneficial to maintain the diversity and stability of bacterial and fungal communities in the goji-alfalfa pattern, while N3 treatment significantly inhibited microbial diversity in the goji berry monoculture pattern. The functional prediction showed that the function of bacteria was mainly amino acid metabolism and carbohydrate metabolism. The ILN1 treatment appeared to facilitate the transformation of fungi to mixed trophic strategies such as *endophyte-plant pathogens*, while the goji berry monoculture pattern tended to rely more heavily on saprophytic nutrition.

**Conclusions:**

In the arid saline-alkali area, the nitrogen reduction management mode of goji-alfalfa intercropping with 150 kg·hm^-^² could effectively reconstruct the microbial community in the root zone of goji berry. It is a suitable cultivation and nitrogen application management mode for the green and sustainable development of goji berry industry in this area.

## 1 Introduction

Goji berry (*Lycium barbarum* L.), a perennial deciduous shrub, belongs to the genus *Lycium* of the Solanaceae family. Its covers many countries and regions in the world (Yan et al., 2025). In arid, semi-arid, and saline–alkaline areas, goji berry plays a dual role in ecological restoration and economic production. Owing to its unique biological characteristics—including tolerance to salinity and alkalinity, poor soil fertility, drought, and wind erosion (Ma et al., 2025), it has become a pioneer species for ecological restoration in arid and saline-alkali regions globally. In regional land reclamation and restoration practices, it is widely adopted and regarded as the preferred species (Yao et al., 2025). Meanwhile, as a world-renowned medicinal and edible homologous crop (Hu et al., 2023; Xiong et al., 2025; Guo et al., 2025), goji berry and its related products have a wide market demand and occupy an important position in the regional economy (Vidović et al., 2022). It has become one of the core pillar industries for rural revitalization and regional economic growth in many planting areas (Cheng et al., 2025).The sustainable development of goji berry cultivation holds significant implications for ecological security and agricultural economic stability in arid regions worldwide (Zhang et al., 2025a).

However, with the advancement of global agricultural modernization, a series of production problems are restricting the green development of various crop industries. Among them, the problem caused by excessive application of nitrogen fertilizer is particularly prominent (Wei et al., 2024; Sun et al., 2025). In order to pursue high yield, the phenomenon of excessive nitrogen fertilizer input is widespread in agricultural production in many parts of the world. This not only results in low nitrogen use efficiency (He et al., 2021), but also leads to substantial nitrogen losses through leaching, volatilization, and other pathways, thereby causing widespread environmental problems such as soil acidification (Ren et al., 2023), eutrophication of aquatic ecosystems, and increased emissions of greenhouse gas (Yin et al., 2017). Moreover, excessive N fertilization disrupts soil microecological balance by inhibiting the activity of beneficial functional microorganisms, including diazotrophs and actinomycetes, and by reducing soil microbial diversity. These changes ultimately impair crop growth, yield, and quality, forming a vicious cycle of “high input–low efficiency–high pollution” (Jiang et al., 2020). Many studies have confirmed the significant regulation of nitrogen application on farmland ecosystems and microbial communities. Wu et al. reported that nitrogen application significantly regulated the infection rate, community diversity and function of arbuscular mycorrhizal fungi (AMF) in the intercropping pattern of spring wheat and pea. The nitrogen application level of 180 kg/hm^2^ was most conducive to improving the symbiotic benefits of AMF and crops (Wu, 2022). Zhuang et al. demonstrated that nitrogen reduction could significantly inhibit the photosynthetic characteristics of vanilla without AMF inoculation, while AMF inoculation could alleviate the adverse effects and improve the nitrogen uptake efficiency and plant key enzyme activities of areca nut, and the combined application of the two could improve the nitrogen use efficiency of intercropping system (Zhuang et al., 2025). Zhao et al. found that, under both zero-nitrogen and conventional nitrogen conditions, the number of bacterial operational taxonomic units (OTUs) in intercropping systems exceeded that in monoculture systems, with a substantially greater increase observed under zero-nitrogen conditions; however, under high nitrogen input, species richness in intercropping systems was markedly lower than that in monoculture (Zhao et al., 2025). These findings indicate that nitrogen, as a fundamental element for plant life activities, is a core regulator of the soil microbial ecosystem. Appropriate nitrogen application meets plant nutrient demands and stimulates beneficial microbial activity, while excessive nitrogen input disrupts microbial community balance. By altering soil physicochemical properties such as carbon-nitrogen ratio and pH, nitrogen application selects for microbial groups with specific functions, ultimately influencing the diversity and functional activity of root zone microbial communities. By changing the physicochemical and chemical properties such as the soil carbon-nitrogen ratio and pH, microbial groups with specific functions are selected, which ultimately affects the diversity and functional activity of microbial communities in the root zone (Abbas et al., 2024).

As a dominant crop in arid saline-alkaline regions worldwide, the growth (Fang et al., 2022), yield, and quality (Zhou et al., 2025) of goji berry are closely associated with the root zone microecological environment (Jiang et al., 2024). As the core component of soil ecosystem, soil microorganisms directly affect the absorption efficiency of soil nutrients by participating in key biological processes such as carbon and nitrogen transformation (Liu et al., 2025), soil nutrient cycling (Zhang et al., 2024) and pathogen inhibition (Zhang et al., 2025b), and regulate its ability to cope with drought, salinity and other stress. At the same time, the climatic characteristics of low precipitation and high evaporation in the global arid areas have aggravated the process of soil salinization. This abiotic stress not only restricts the development of crop roots, but also significantly inhibits soil microbial activity, which further aggravates the contradiction between industrial development and ecological protection (Mi et al., 2020). At present, the main producing areas of goji berry are generally facing problems such as increased soil salinization, imbalance of root zone microecology, and low nitrogen fertilizer utilization efficiency. How to solve these difficulties through scientific field management measures and realize the green, efficient, and sustainable development of the goji berry industry has become a common topic in the field of agricultural research in arid areas around the world.

Against this background, intercropping is regarded as one of the effective ways to optimize the microecology of the crop root zone and solve the agricultural dilemma in arid and saline-alkali areas because of its complementary resources and microenvironment regulation advantages. Extensive studies have been conducted worldwide to investigate the effects of intercropping on soil microbial communities across a range of cropping patterns, including tea plantations, orchards, maize, wheat, and other major crops. A general consensus has emerged that intercropping exert more favorable effects on root zone microecological conditions than monoculture. Ding et al. reported that nitrogen uptake in a pepper–maize intercropping pattern was 28% higher than that in pepper monoculture (Ding et al., 2020). Liang et al. demonstrated that maize–alfalfa intercropping significantly enhanced soil microbial diversity (Liang et al., 2025). Yin et al. found that cucumber–celery intercropping enriched specific amplicon sequence variants (ASVs) while simultaneously suppressing the proliferation of pathogenic fungi (Yin et al., 2025). Studies have shown that grass-legume intercropping systems can increase the abundance and activity of beneficial root zone bacteria by virtue of the interaction between the nitrogen fixation characteristics of legumes and root exudates (Maitra and Ray, 2019). Intercropping goji berry with the leguminous crop alfalfa can supplement soil nitrogen via symbiotic nitrogen fixation by alfalfa rhizobia. In addition, organic acids, flavonoids, and other compounds released by alfalfa roots can regulate the root zone carbon-to-nitrogen ratio, thereby providing more diverse and accessible nutrient substrates for soil microorganisms and promoting the proliferation of beneficial microbial populations (Wang et al., 2021). In summary, intercropping can improve soil microbial community structure, enhance microbial diversity and activity, increase the number of beneficial microorganisms, and reduce that of harmful microorganisms, thereby promoting soil nutrient cycling (Wang, 2025) and improving crop resource use efficiency (Gitari et al., 2018).

At present, an increasing number of studies have examined the effects of cropping patterns and nitrogen application rates on root zone soil microbial communities. Most of these investigations have focused on crops such as maize, soybean, wheat, sorghum, tea plantations, and pea, and have largely been conducted in grain-based or conventional agroecosystems. But studies addressing goji-alfalfa intercropping patterns in saline-alkaline soils remain limited. Jingtai County in Gansu Province represents a typical arid saline-alkaline region in China and has long been confronted with severe challenges, including low precipitation, high evaporation rates, and pronounced soil salinization (Zheng, 2024). Moreover, goji berry cultivation in this region is commonly characterized by excessive nitrogen inputs, low nitrogen use efficiency, and root zone microecological imbalance under traditional monoculture patterns (Yin, 2022). These constraints closely mirror the broader challenges facing goji berry production in arid regions worldwide. Therefore, this study was conducted in Jingtai County using goji berry as the target crop and incorporating two cropping patterns and four nitrogen application rates to study the following core issues: (1) To explore the effects of planting patterns and nitrogen application levels on the microbial community in the root zone of goji berry. (2) To reveal the differential characteristics of microbial abundance and community structure in the root zone of goji berry under different cropping patterns, nitrogen application rates, and their interactions; to clarified the response patterns of microbial α-diversity and β-diversity to the combination of cropping patterns and nitrogen application levels, and to identified the correlation mechanism between microbial community functions and integrated management practices. (3) To identify the most suitable combination of cropping pattern and nitrogen application rate for regulating rootzone ecosystem function in arid saline-alkaline regions. It provides theoretical support and practical reference for the micro-ecological regulation, intercropping mode optimization and nitrogen reduction and efficiency production of goji berry root zone in arid saline-alkali areas, and then promotes the green, efficient and sustainable development of goji berry industry.

## 2 Materials and methods

### 2.1 Description of the experimental site

The field experiment was conducted from May to October 2025 at the Irrigation Experimental Station of the Jingtaichuan Electric Pumped Power Water Resources Utilization Center, Gansu Province, China (37°23′N, 104°08′E; mean elevation 1563 m). The experimental site has a typical temperate continental arid saline-alkali climate. The average annual solar radiation in the test area is 6.18 × 10^5^ J·cm^-2^, with annual sunshine hours are about 2652 h, the frost-free period is about 191 d, and the average annual evaporation is 2761 mm. During the 2025 trial period, the daily average temperature was 15.07 °C, with total effective precipitation of 317.92 mm. Meteorological observation data were collected in real time by a Davis automatic weather station installed in the experimental field. The soil at the experimental site is classified as sandy loam, with a bulk density of 1.66 g·cm^-^³ and a field water-holding capacity of 24.13% (volumetric water content). In the 0–60 cm soil layer, the contents of soil organic matter, total nitrogen, total phosphorus, and total potassium contents were 13.20, 1.62, 1.32, and 34.03 g·kg^-^¹, respectively. Available ammonium nitrogen, available phosphorus, and available potassium contents were 74.51, 26.31, and 173 mg·kg^-^¹, respectively. The soil pH was 8.11, with a total salt content of 7.38 g·kg^-^¹, a sodicity (alkalization degree) of 26.56%, and an electrical conductivity (EC) of 2.34 dS·m^-^¹.

### 2.2 Experimental design

The experiment was arranged in a randomized complete block design. The goji berry cultivar “Ningqi No. 5” was used as the test material. Based on previous studies (Lv et al., 2025; Wang et al., 2024), nitrogen application rates and cropping patterns were selected as the experimental factors. Two cropping patterns (goji berry monoculture and goji–alfalfa intercropping) were assigned as main plots, while four nitrogen application levels (high, medium, low, and zero nitrogen) were allocated to subplots. All treatments were irrigated via drip irrigation pattern under full irrigation conditions, with soil moisture at 75–85% of field capacity (θf). To ensure the stability of the microbial habitat, soil volumetric water content in the 0–20 cm soil layer (matching the rhizosphere soil sampling depth) was monitored every 7 days using a PICO-BT TRIME-TDR instrument (IMKO, Germany). Additional measurements were conducted immediately before and after irrigation or rainfall events, and sensor data were periodically calibrated using the oven-drying method to ensure accuracy. This monitoring regime effectively captured the soil moisture dynamics critical for microbial growth and activity throughout the experimental period. Drip irrigation lines were installed on both the northern and southern sides of each goji berry plant at a distance of 0.15 m from the trunk. A total of eight treatments were established, with three replicates per treatment. Detailed descriptions and codes of the experimental treatments are presented in **Table 1**. Each experimental plot covered an area of 76.5 m² (10.2 m × 7.5 m). In the monoculture pattern, each plot contained 20 goji berry plants arranged in a 4 × 5 configuration, with a planting density of 1.5 m × 3.0 m. In the goji–alfalfa intercropping pattern, the planting density of goji berry was identical to that in monoculture. Five rows of alfalfa (Medicago sativa L., cultivar “Longdong Alfalfa”) were sown in strips at a distance of 0.9 m from the goji berry trunks, with a seeding rate of 13 kg·hm^-^². The intercropping pattern was established in 2021. Nitrogen fertilizer (urea, 46% N) was applied via a Venturi injector through the drip irrigation pattern and split into the vegetative growth stage, full flowering stage, and peak fruiting stage at a ratio of 6:2:2. The actual nitrogen application rates for each treatment at different growth stages were as follows: N0 (0 kg·hm^-^² total: 0, 0, 0 kg·hm^-^²); N1 (150 kg·hm^-^² total: 90, 30, 30 kg·hm^-^²); N2 (300 kg·hm^-^² total: 180, 60, 60 kg·hm^-^²); N3 (450 kg·hm^-^² total: 270, 90, 90 kg·hm^-^²), corresponding to the vegetative growth, full flowering, and peak fruiting stages, respectively. Phosphorus fertilizer (calcium superphosphate, 12% P) and potassium fertilizer (potassium chloride, 60% K) were applied once as basal fertilizer during the vegetative growth stage. All other field management practices, including pruning and pest and disease control, followed local conventional farming practices.

**Table 1:** Experimental treatments and their codes.

Cropping pattern	Nitrogen application rate (pure N)	Code
goji berry monoculture (ML)	High nitrogen(450 kg·hm-2)	MLN3
	Medium nitrogen(300 kg·hm-2)	MLN2
	Low nitrogen(150 kg·hm-2)	MLN1
	No nitrogen application(0 kg·hm-2)	MLN0
goji- alfalfa intercropping (IL)	High nitrogen(450 kg·hm-2)	ILN3
	Medium nitrogen(300 kg·hm-2)	ILN2
	Low nitrogen(150 kg·hm-2)	ILN1
	No nitrogen application(0 kg·hm-2)	ILN0

### 2.3 Sample collection and measurements

#### 2.3.1 Soil sampling

Soil sampling was conducted in the root zone soil of goji berry plants at the full fruiting stage (mid-August). In each plot, three goji berry plants with uniform growth were randomly selected for sampling, while plants located at plot borders were excluded to minimize edge effects. Four symmetrical sampling points were evenly arranged around goji berry plants by the ring sampling method. With each goji berry plants as the center, surface withered branches and leaves were removed in the root zone about 10 cm away from the roots. The soil samples from the 0–20 cm soil layers were taken at four sampling positions with a sterile stainless-steel soil auger. The soil samples from the four sampling positions were mixed in a sterile environment and divided to 200 g using the quartering method. The soil samples were divided into two parts, which were transported back to the laboratory at low temperature (4 °C) within 24 h. One part was stored at -80 °C for high-throughput sequencing, and the other was temporarily stored in a refrigerator at 4 °C for the determination of culturable microbial abundance (Lian, 2024).

#### 2.3.2 Determination of culturable microbial abundance and soil physicochemical properties

The abundances of culturable bacteria, actinomycetes, and fungi were determined using the serial dilution plate count method. It should be noted that this method only quantifies culturable microorganisms, and more than 99% of soil microorganisms are unculturable under standard laboratory conditions; thus, the results cannot represent the total soil microbial biomass (Na et al., 2021). Fresh soil samples temporarily stored at 4 °C were transferred to a laminar flow hood and allowed to equilibrate to room temperature for about 2 h. The soil samples were then thoroughly homogenized, and eight serial tenfold dilutions were prepared using sterile distilled water, corresponding to dilution levels of 10^-^¹, 10^-^², 10^-^³, 10^-4^, 10^-5^, 10^-6^, 10^-7^, and 10^-8^.

**Figure 1:**
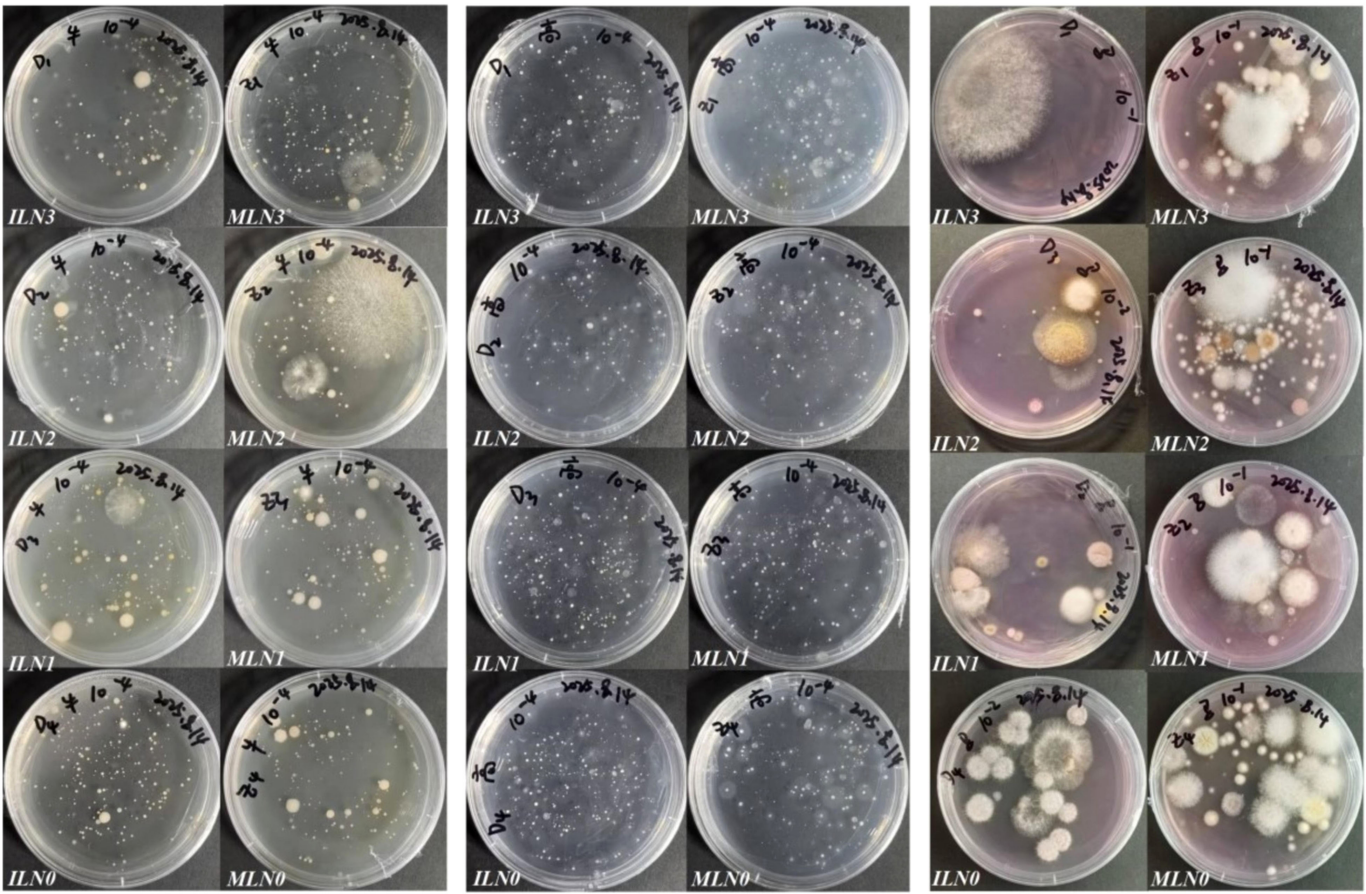
Culturable microbial colonies on culture media (left: culturable bacteria; middle: culturable actinomycetes; right: culturable fungi).

For bacterial enumeration, beef extract–peptone agar was used, and plates were incubated at 30 °C for 1–2 days in an incubator. Actinomycetes were enumerated using Gause’s No.1 medium, with incubation at 28 °C for 5–7 days. Culturable fungal abundance was determined using Martin’s medium, which was supplemented with 1% streptomycin solution and 1% Bengal red solution during preparation to suppress bacterial growth, regulate fungal growth, and improve colony visualization. Fungal plates were incubated at 28 °C for 3–5 days (Ma and Fan, 2025; Qu et al., 2021). After incubation, plates with appropriate dilution levels were selected for colony counting. Specifically, dilutions of 10^-4^–10^-5^ were used for bacteria and actinomycetes, while dilutions of 10^-^¹–10^-^³ were used for fungi. For reliable enumeration, plates with 30–300 colonies were considered suitable for bacteria and actinomycetes, whereas those with 10–100 colonies per plate were deemed appropriate for fungi (**Figure 1**). Care was taken to avoid counting contaminated colonies. Three replicate plates were prepared for each dilution to minimize random error. Culturable microbial abundance was expressed as colony-forming units per gram of fresh soil (CFU·g^-^¹ fresh soil) and calculated using Equation 1 as follows:

(1) $$N=\frac{\bar{C}\times D}{V}$$

*N*: culturable microbial abundance, CFU·g^-^¹; $\bar{C}$: mean number of colonies per plate; *D*: dilution factor; *V*: inoculation volume, mL.

Soil pH and electrical conductivity (EC) were measured using a pH meter and a conductivity meter (soil-water ratio 1:5), and soil total nitrogen (total N) content was determined using the Kjeldahl method.

#### 2.3.3 High-throughput sequencing of root zone soil microorganisms

High-throughput sequencing of bacterial and fungal communities in root zone soil samples was conducted by Novogene Bioinformatics Technology Co., Ltd. (Tianjin, China). The V4 region of the bacterial 16S rRNA gene was amplified using the primer pair 515F (5′-GTGCCAGCMGCCGCGGTAA-3′) and 806R (5′-GGACTACHVGGGTWTCTAAT-3′), while the ITS2 region of the fungal internal transcribed spacer was amplified using the primer pair ITS3 (5′-GCATCGATGAAGAACGCAGC-3′) and ITS4 (5′-TCCTCCGCTTATTGATATGC-3′). Sequencing libraries were constructed based on the amplified regions and subsequently sequenced on an Illumina platform following the manufacturer’s standard protocols.

#### 2.3.4 Data processing and statistical analysis

Bioinformatic analyses were performed using the Novogene NovoMagic Microbial Analysis Platform (https://magic.novogene.com). Alpha diversity indices were calculated in QIIME 2, including Chao1, Good’s coverage, Observed Features, Pielou’s evenness (Pielou_e), Shannon, and Simpson indices, to characterize species richness, diversity, and evenness under different treatments, as well as to visually assess sequencing depth and data sufficiency. Across all treatments, Good’s coverage values for soil bacterial communities were ≥ 0.994, while those for fungal communities reached 1.000, indicating sufficient sequencing depth and high reliability of the microbial community data. Principal coordinates analysis (PCoA) and redundancy analysis (RDA) were performed and visualized in R software (v4.0.0, https://www.r-project.org/) based on Bray–Curtis distances (PCoA; NMDS; RDA). Microbial community heatmaps were also constructed in R software Analysis of similarities (ANOSIM) was applied to evaluate the significant differences in microbial β-diversity among treatments. Data collation was conducted using Microsoft Excel 2019, statistical significance testing was performed using SPSS 26.0 at a significance level of *P* < 0.05, and relative abundance histograms were generated using Origin 2021.

## 3 Results

### 3.1 Effects of cropping pattern and nitrogen application rate on root zone soil microbial abundance and community composition of goji berry

#### 3.1.1 Culturable microbial abundance

Cropping pattern, nitrogen application rate, and their interaction exerted highly significant effects on soil microbial culturable abundance (*P* < 0.01; **Table 2**). Compared with goji berry monoculture (ML) at the same nitrogen application level, the regulation of goji-alfalfa intercropping (IL) on culturable microbial abundance was characterized by guild specificity and nitrogen application dependence: culturable bacterial abundance was increased by 35.12%, 18.29%, and 31.48% under IL at N0, N1, and N2 levels, respectively, compared with ML, while decreased by 28.52% at the N3 level, showing a response pattern of promotion under low nitrogen and inhibition under high nitrogen. Culturable actinomycete abundance was increased by 6.98% and 8.49% under IL at N0 and N1 levels respectively compared with ML only, while decreased by 17.75% and 36.52% at N2 and N3 levels, indicating a better adaptability to low-nitrogen environment in the intercropping system. Culturable fungal abundance was inhibited by intercropping at all nitrogen application levels, with the strongest inhibitory effect at N2 level (a decrease of 71.03%) and the weakest at N1 level (a decrease of 15.29%), and the decreases at N0 and N3 levels were 49.66% and 59.62% respectively compared with ML, which reflected the selective regulation of intercropping on fungal community. The effect of nitrogen application rate on culturable microbial abundance was dependent on cropping pattern: under the goji-alfalfa intercropping pattern, culturable bacterial abundance reached the maximum at N2 treatment, increasing by 62.33%, 20.15% and 14.08% compared with N3, N1 and N0 treatments respectively; culturable actinomycete abundance was the highest at N1 treatment, increasing by 78.63%, 25.63% and 6.36% compared with N3, N2 and N0 treatments respectively; while culturable fungal abundance was the lowest at N3 treatment, decreasing by 44% compared with N0 treatment. Under the goji berry monoculture pattern, culturable bacterial abundance increased progressively with the increase of nitrogen application rate and peaked at N3 treatment, increasing by 13.31%, 22.49% and 32.85% compared with N2, N1 and N0 treatments respectively; both actinomycete and culturable fungal abundances peaked at N2 treatment, with culturable actinomycete abundance increasing by 9.74%, 4.99% and 10.12% compared with N3, N1 and N0 treatments respectively, and culturable fungal abundance increasing by 90.38%, 132.94% and 32.89% compared with the corresponding treatments respectively. Overall, a differentiated nitrogen response pattern was presented that intercropping system promoted the proliferation of beneficial culturable microorganisms under low nitrogen conditions, while monoculture system was more dependent on medium and high nitrogen input to maintain culturable microbial abundance.

**Table 2:** Abundances of bacteria, actinomycetes and fungi under different treatments (fresh soil).

Cropping pattern	Nitrogen application Rate	Bacterial abundance (×10^6^ CFU/g)	Actinomycete abundance (×10^5^ CFU/g)	Fungal abundance (×10^3^ CFU/g)
ML	N3	8.17 ± 0.32b	5.75 ± 0.16c	2.08 ± 0.07c
	N2	7.21 ± 0.26c	6.31 ± 0.30ab	3.96 ± 0.13a
	N1	6.67 ± 0.33cd	6.01 ± 0.22bc	1.70 ± 0.06d
	N0	6.15 ± 0.28de	5.73 ± 0.24c	2.98 ± 0.11b
IL	N3	5.84 ± 0.21e	3.65 ± 0.18e	0.84 ± 0.03f
	N2	9.48 ± 0.49a	5.19 ± 0.23d	1.46 ± 0.06e
	N1	7.89 ± 0.22b	6.52 ± 0.30a	1.44 ± 0.07e
	N0	8.31 ± 0.38b	6.13 ± 0.24abc	1.50 ± 0.05e
	Analysis of variance			
	C	**	**	**
	N	**	**	**
	C×N	**	**	**

C represents the cropping pattern, N represents the nitrogen application level, and C×N represents their interaction. Different lowercase letters indicate differences among treatments within the same year. ** indicates a highly significant difference (*P* < 0.01). CFU is the abbreviation for colony-forming units, which is the standard unit for quantifying soil microbial abundance.

#### 3.1.2 Bacterial community structure at the phylum level

**Figure 2:**
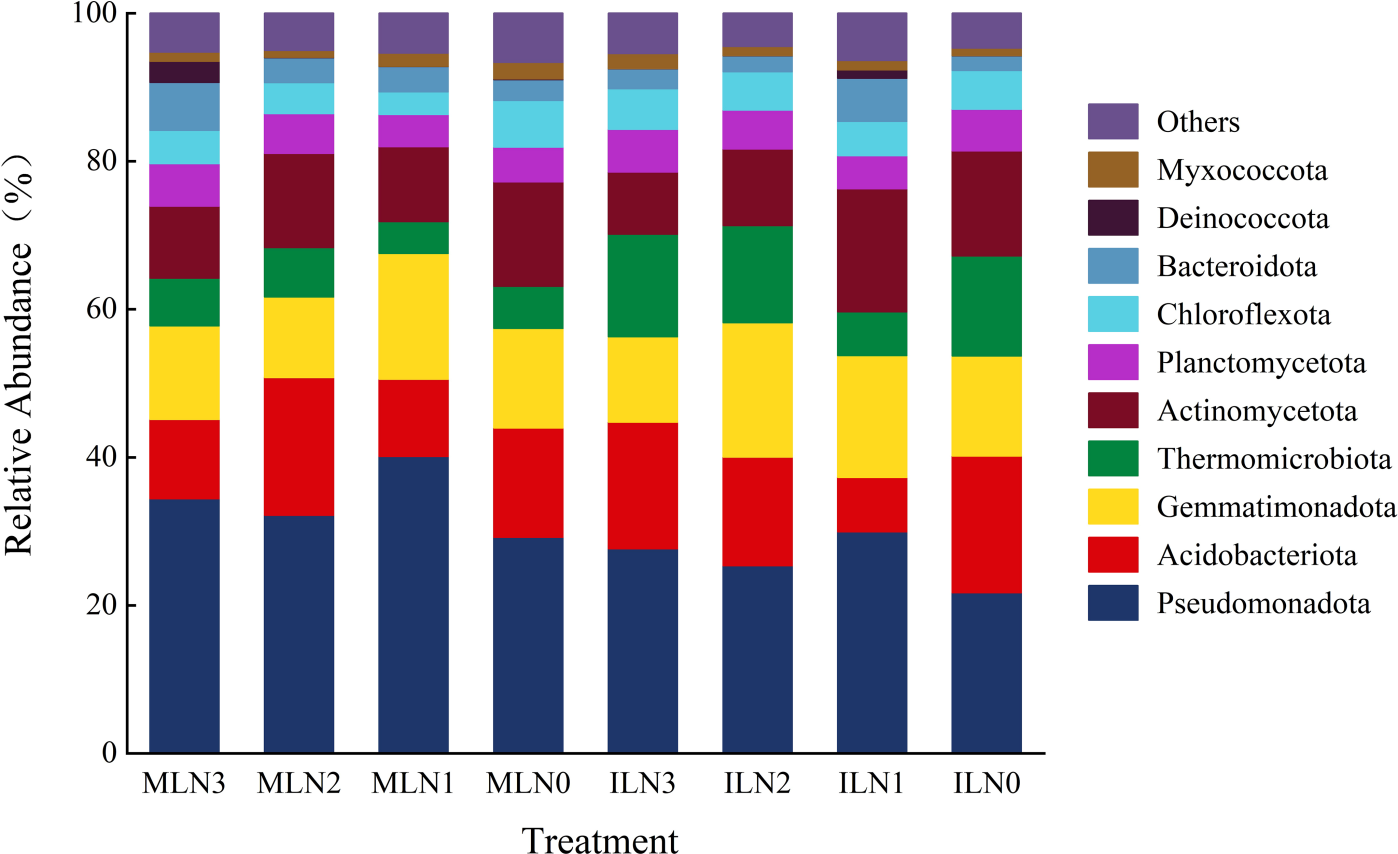
Community composition and relative abundance of soil bacteria at the phylum level under different treatments. IL: Goji-alfalfa intercropping; ML: Goji berry monoculture; N0-N3: Nitrogen application rates of 0, 150, 300, 450 kg·hm^-^², respectively. The top 10 phyla with the highest relative abundance in all treatments were selected, and the remaining taxa were classified as “Others”.

As shown in **Figure 2**, the dominant bacterial phyla were generally consistent between the goji–alfalfa intercropping and goji berry monoculture patterns, including *Pseudomonadota*, *Acidobacteriota*, *Gemmatimonadota*, *Thermomicrobiota*, *Actinomycetota*, and *Planctomycetota*. Together, these six phyla accounted for 84.78% and 83.61% of the total relative abundance in the intercropping and monoculture patterns, respectively. However, pronounced differences in relative abundance were primarily observed for *Pseudomonadota* and *Thermomicrobiota*. Specifically, the relative abundance of *Pseudomonadota* in the intercropping pattern was significantly lower than that in the monoculture pattern by 22.98% (*P* < 0.05), where it constituted the dominant phylum. In contrast, the relative abundance of *Thermomicrobiota* was significantly higher in the intercropping pattern than in the monoculture pattern, increasing by 101.04% (*P* < 0.05), and thus representing a characteristic dominant phylum under intercropping conditions. With respect to nitrogen application rate, under the goji–alfalfa intercropping pattern, the relative abundances of *Pseudomonadota* and *Actinomycetota* peaked under the N1 treatment (29.95% and 16.64%, respectively), representing increases of 8.28%, 18.10%, and 37.83% for *Pseudomonadota* and 98.10%, 60.93%, and 17.10% for *Actinomycetota* compared with the N3, N2, and N0 treatments, respectively. Under the goji berry monoculture pattern, *Pseudomonadota* exhibited the highest relative abundance under the N1 treatment (40.13%), increasing by 16.66%, 24.70%, and 37.43% relative to the N3, N2, and N0 treatments, respectively. In contrast, *Actinomycetota* reached its maximum relative abundance under the N0 treatment (14.11%), exceeding that under the N3, N2, and N1 treatments by 45.02%, 11.01%, and 39.29%, respectively.

#### 3.1.3 Bacterial community structure at the genus level

**Figure 3:**
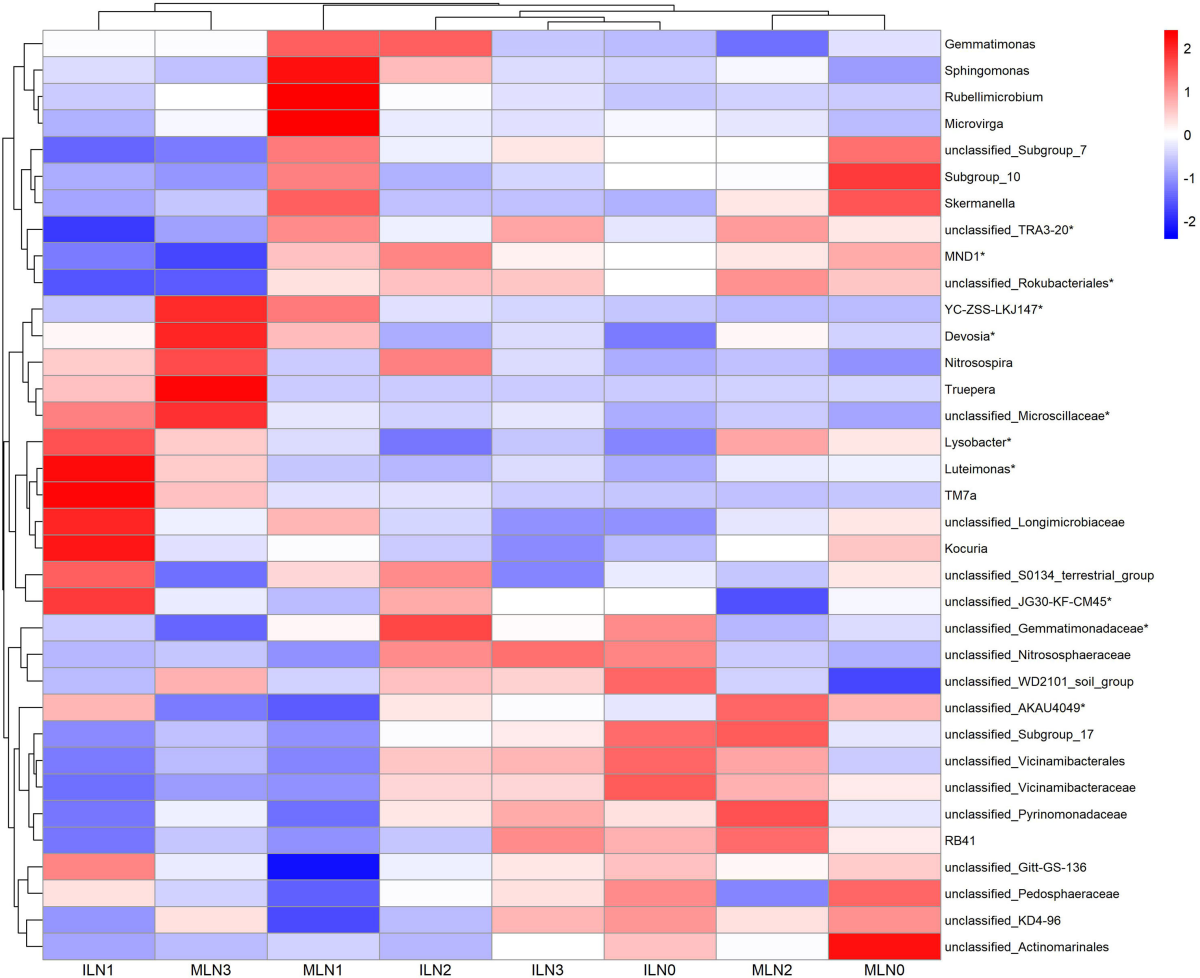
Heatmap of the top 35 dominant bacterial genera under different treatments (based on relative abundance). IL: Goji-alfalfa intercropping; ML: Goji berry monoculture; N0-N3: Nitrogen application rates of 0, 150, 300, 450 kg·hm^-^², respectively. The color gradient represents the relative abundance of bacterial genera (red: high abundance, blue: low abundance).

Based on the phylum-level community structure analysis, the top 35 dominant genera by relative abundance were further analyzed to clarify differences in microbial community composition among treatments. As visualized in the heatmap (**Figure 3**), the relative abundances of dominant bacterial genera showed distinct separation patterns between cropping patterns and across nitrogen application rates. Samples from the intercropping (IL) and monocropping (ML) systems were generally separated into different clusters, indicating that cropping pattern exerted a considerable effect on the bacterial community composition at the genus level. Within the intercropping system, the ILN1 treatment maintained relatively high abundances of several key genera, including *Luteimonas* (2.26), *TM7a* (2.27), *Kocuria* (2.16), *unclassified_Longimicrobiaceae* (1.98), and *Lysobacter* (1.62), which were notably higher than those in other intercropping treatments (ILN0, ILN2, ILN3). Statistical analysis confirmed that the relative abundances of *Luteimonas*, *Lysobacter*, and *unclassified_JG30-KF-CM45* in ILN1 were significantly different from those in other treatments (*P* < 0.05). In contrast, the ILN0 and ILN3 treatments were dominated by different taxa, such as *unclassified_Vicinamibacteraceae* (1.53) and *unclassified_WD2101_soil_group* (1.43), respectively. Under the monocropping system, the community structure of dominant genera also exhibited clear variation with nitrogen application rate. The MLN1 treatment was characterized by high abundances of *Rubellimicrobium* (2.42), *Microvirga* (2.41), and *Sphingomonas* (2.21), while MLN0 and MLN2 showed enrichment of *unclassified_Actinomarinales* (2.19) and *unclassified_Pyrinomonadaceae* (1.64), respectively. The relative abundances of *YC-ZSS-LKJ147* and *unclassified_Microscillaceae* in MLN3 were significantly higher than those in other monocropping treatments (*P* < 0.05).

#### 3.1.4 Fungal community structure at the phylum level

**Figure 4:**
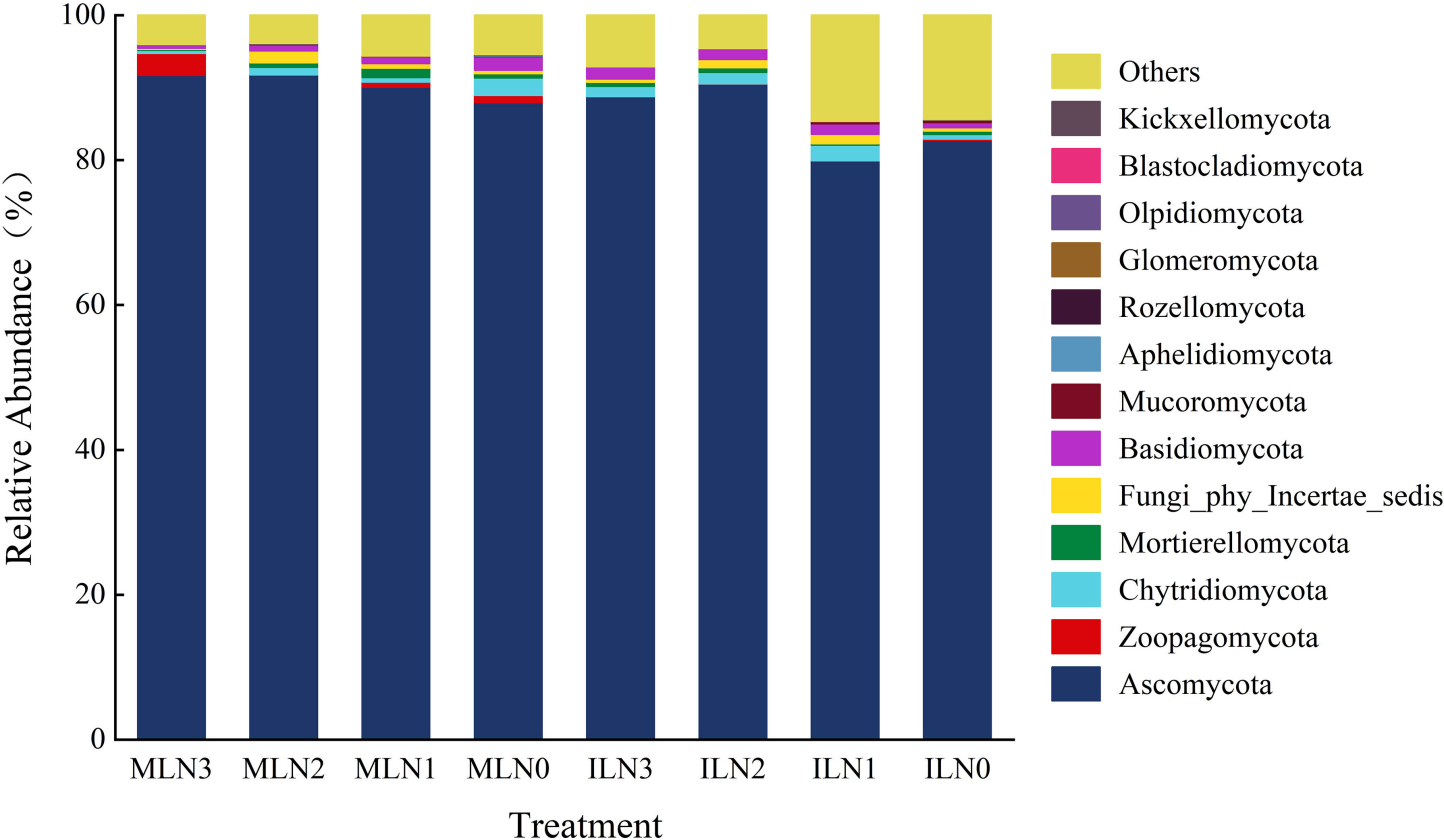
Community composition and relative abundance of soil Fungi at the phylum level under different treatments. IL: Goji-alfalfa intercropping; ML: Goji berry monoculture; N0-N3: Nitrogen application rates of 0, 150, 300, 450 kg·hm^-^², respectively. The top 10 phyla with the highest relative abundance in all treatments were selected, and the remaining taxa were classified as “Others”.

As shown in **Figure 4**, the dominant fungal phylum was identical in the goji-alfalfa intercropping and goji berry monoculture patterns, with Ascomycota being the absolute dominant phylum, whose relative abundances were 85.48% and 90.37% in the intercropping and monoculture patterns, respectively. Under the intercropping pattern, the relative abundances of Basidiomycota and the “Others” group were increased by 25.00% and 114.32% compared with those under the monoculture pattern, while that of *Zoopagomycota* was significantly decreased by 95.80% (*P* < 0.05) relative to the monoculture pattern, showing a guild-specific regulatory characteristic. The regulation of nitrogen application rate on fungal phylum-level abundance was dependent on cropping patterns, with the two patterns exhibiting distinctly different nitrogen response laws of fungi: *Ascomycota* had the highest abundance at N2 treatment in both patterns, with 90.51% and 91.77% in the intercropping and monoculture patterns, respectively. In the intercropping pattern, its abundance was increased by 13.28% and 9.38% compared with N1 and N0 treatments, respectively, and in the monoculture pattern, it was increased by 1.89% and 4.38% compared with the corresponding treatments, respectively. In the goji-alfalfa intercropping pattern, *Chytridiomycota* reached a peak abundance of 2.17% at N1 treatment, increasing by 51.75%, 35.63% and 255.74% compared with N3, N2 and N0 treatments, respectively, and Basidiomycota peaked at 1.62% at N3 treatment, increasing by 13.29%, 12.50% and 128.17% compared with N2, N1 and N0 treatments, respectively. In the goji berry monoculture pattern, both *Chytridiomycota* and *Basidiomycota* had the highest abundances at N0 treatment, being 2.41% and 1.84%, respectively. Among them, the abundance of *Chytridiomycota* was increased by 460.47%, 288.71% and 129.52% compared with N3, N2 and N1 treatments, respectively, and that of *Basidiomycota* was increased by 268.00%, 85.86% and 119.05% compared with the corresponding treatments, respectively.

#### 3.1.5 Fungal community structure at the genus level

**Figure 5:**
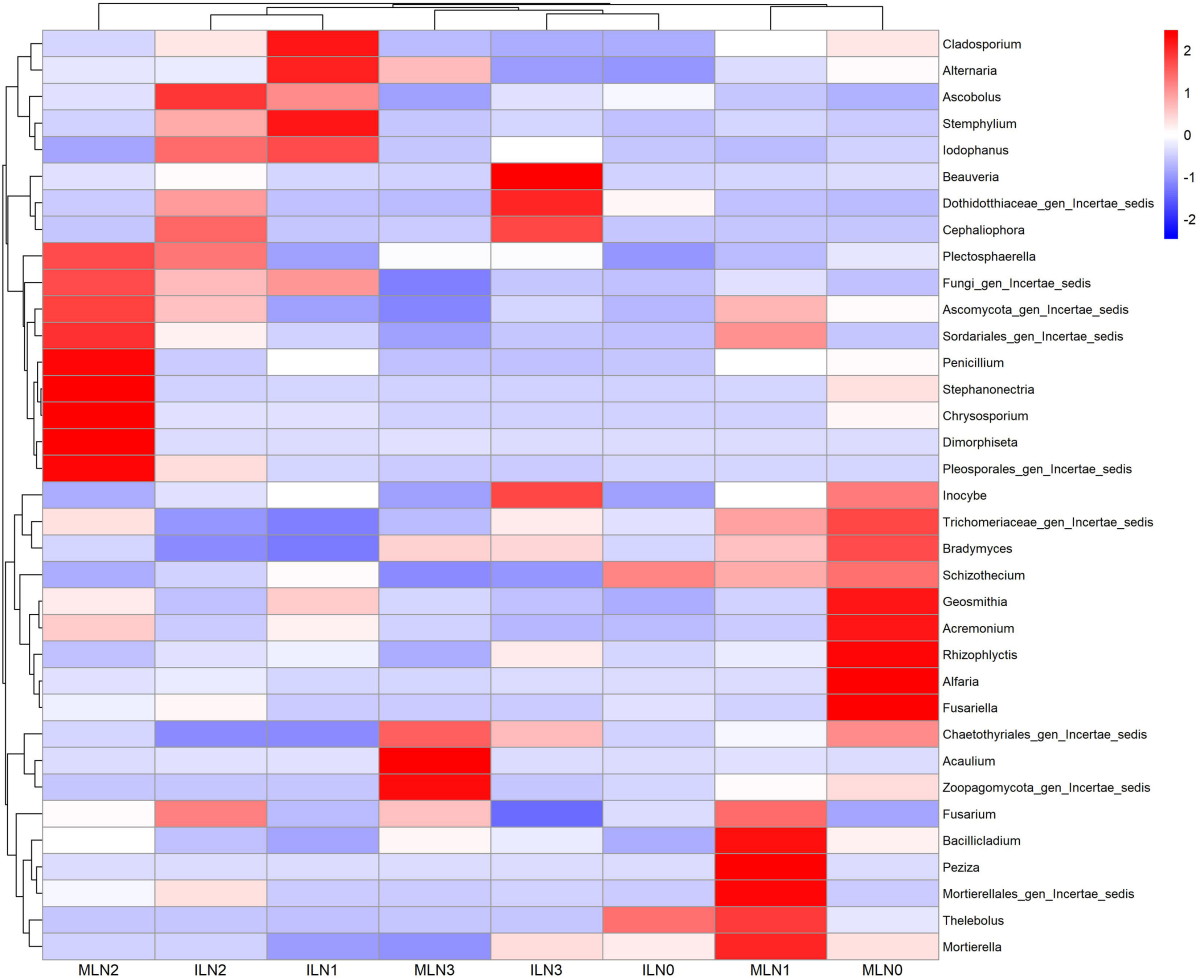
Heatmap of the top 35 dominant fungal genera under different treatments (based on relative abundance). IL: goji-alfalfa intercropping; ML: goji berry monoculture; N0-N3: Nitrogen application rates of 0, 150, 300, 450 kg·hm^-^², respectively. The color gradient represents the relative abundance of fungal genera (red: high abundance, blue: low abundance).

Based on the phylum-level community structure analysis, the top 35 dominant genera by relative abundance were further analyzed to clarify differences in fungal community composition among treatments. As visualized in the heatmap (**Figure 5**), the relative abundances of dominant fungal genera presented clear separation trends between cropping patterns and across nitrogen application rates, though the inter-group differentiation was less pronounced compared with that of bacteria. Samples from the intercropping (IL) and monocropping (ML) systems tended to cluster into different groups, implying a potential regulatory effect of cropping pattern on the fungal community composition at the genus level. Within the intercropping system, the ILN1 treatment exhibited relatively higher abundances of several saprophytic and plant-associated genera, including *Cladosporium* (2.21), *Alternaria* (2.09), *Stemphylium* (2.19), *Ascobolus* (1.13), and *Iodophanus* (1.73). These genera showed an enrichment trend in ILN1 relative to ILN0, ILN2, and ILN3 treatments, and statistical analysis revealed a marginally significant response (0.05 < *P* < 0.1) of *Ascobolus* (*P* = 0.059) and *Iodophanus* (*P* = 0.063) to the combined treatments of cropping pattern and nitrogen application rate. In contrast, the ILN2 treatment was dominated by *Ascobolus* (1.93), while the ILN3 treatment showed the highest abundance of *Beauveria* (2.44), though these differences were not statistically significant (*P* > 0.1). Under the monocropping system, the distribution of dominant fungal genera varied with nitrogen application rate. The MLN1 treatment was characterized by high abundances of *Bacillicladium* (2.27), *Fusarium* (1.40), and *Mortierellales_gen_Incertae_sedis* (2.36), among which *Bacillicladium* showed a marginally significant difference (0.05 < *P* < 0.1) across treatments (*P* = 0.068). The MLN0 treatment was enriched with *Alfaria* (2.47) and *Fusariella* (2.41), while the MLN2 treatment had the highest abundances of *Stephanonectria* (2.38), *Penicillium* (2.37), and *Chrysosporium* (2.42). No statistically significant differences (*P* > 0.1) were detected for the majority of dominant fungal genera across the monocropping treatments. Collectively, the distribution of dominant fungal genera at the genus level was affected by both cropping pattern and nitrogen application rate, but the regulatory effect was relatively weak compared with that on bacteria. Only a few genera (i.e., *Ascobolus*, *Iodophanus*, *Bacillicladium*) showed marginally significant enrichment trends (0.05 < *P* < 0.1) to the combined treatments, while the relative abundances of most dominant fungal genera exhibited no significant statistical differentiation among all treatments (*P* > 0.1). The intercropping pattern combined with moderate nitrogen application (ILN1) still formed a distinctive fungal community structure, which might be related to the subtle changes in the soil microenvironment under this management practice.

### 3.2 Effects of cropping pattern and nitrogen application rate on soil physicochemical properties and environmental driving factors of microbial community

Planting patterns and nitrogen application rate had significant effects on soil pH, electrical conductivity (EC) and total nitrogen content (TN) (*P* < 0.01). The interaction effect test showed that the contents of EC and total nitrogen were significantly regulated by the interaction between planting patterns and nitrogen application rate (*P* < 0.01), while pH showed no significant interaction effect (*P* > 0.05). Under the same nitrogen application conditions, the soil pH of the intercropping treatment (IL) was significantly lower than that of the monoculture treatment (ML), and the EC was also significantly reduced. These results indicated that alfalfa intercropping could effectively alleviate soil alkalization and reduce salt accumulation, thus improving saline-alkali soil conditions; Under the same planting pattern, with the increase of nitrogen application rate, the soil pH decreased significantly, which was mainly related to the release of H^+^ in the nitrification process of urea, which led to soil acidification. EC increased with the increase of nitrogen application rate, which was closely related to the continuous accumulation of soluble salt ions brought by fertilizer. The total nitrogen content increased with the increase of nitrogen application level. Based on the changes of various indicators, the EC under ILN1 treatment was the lowest (1.81 dS·m^-^¹), the degree of salt stress was light, and the pH was 7.98, which was in the weak alkaline range suitable for microbial growth. At the same time, the total nitrogen content was moderate, and the rhizosphere environment with low salt, suitable alkali and stable nitrogen supply was formed as a whole, which provided good environmental conditions for the stable development of microbial community (**Table 3**).

**Table 3:** Soil pH, EC, and TN under different cropping patterns and nitrogen application rates.

Cropping pattern	Nitrogen application rate	PH	EC (dS·m^-^¹)	TN (g·kg^-^¹)
IL	N3	7.87 ± 0.03e	2.27 ± 0.05e	1.40 ± 0.02a
	N2	7.94 ± 0.02d	2.21 ± 0.04f	1.30 ± 0.03b
	N1	7.98 ± 0.02d	1.81 ± 0.03d	1.22 ± 0.02d
	N0	8.05 ± 0.04c	1.98 ± 0.02c	1.05 ± 0.03e
ML	N3	8.11 ± 0.03b	2.72 ± 0.03a	1.31 ± 0.02bc
	N2	8.15 ± 0.02b	2.55 ± 0.04b	1.26 ± 0.02c
	N1	8.20 ± 0.03a	2.49 ± 0.05b	1.06 ± 0.03e
	N0	8.22 ± 0.04a	2.27 ± 0.03c	0.99 ± 0.02e
	Analysis of variance			
	C	**	**	**
	N	**	**	**
	C×N	ns	**	**

C represents the cropping pattern, N represents the nitrogen application level, and C×N represents their interaction. Different lowercase letters indicate differences among treatments within the same year. ** indicates a highly significant difference (*P* < 0.01), and ns indicates no significant difference. EC: electrical conductivity; TN: Total nitrogen.

**Figure 6:**
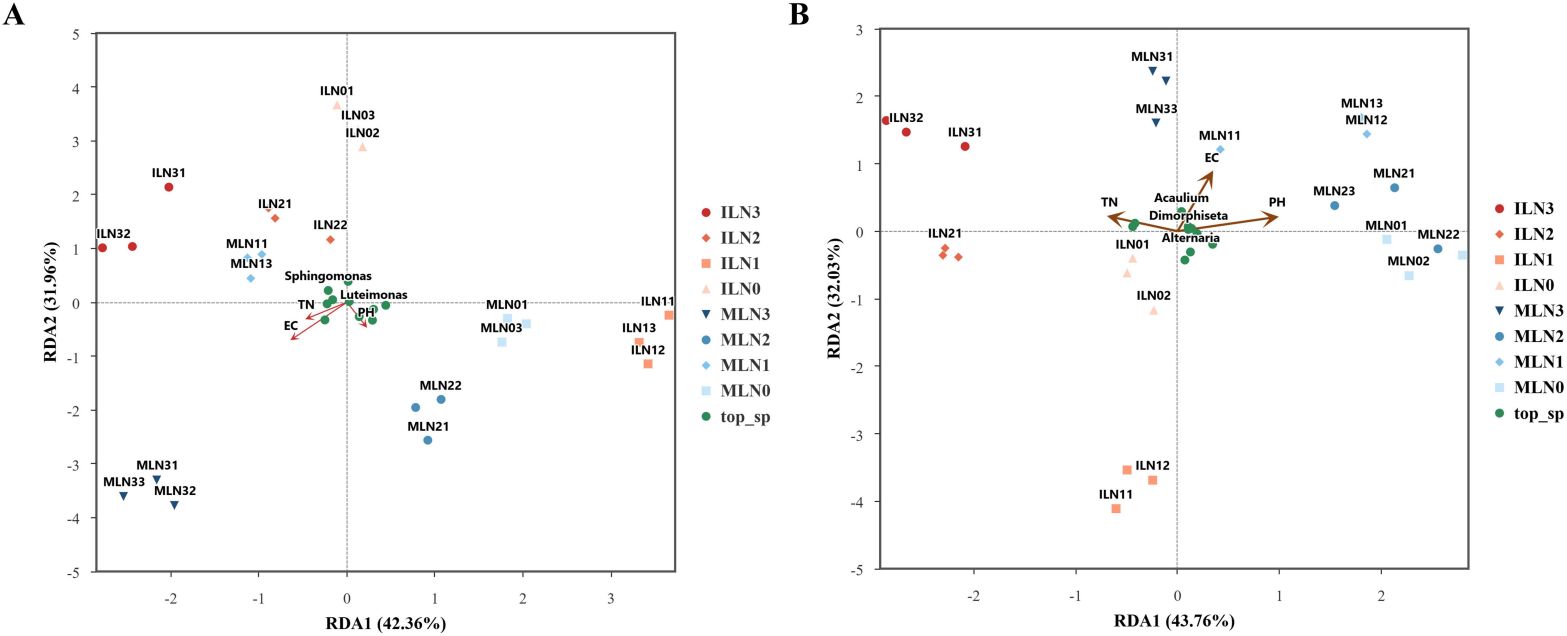
Redundancy analysis (RDA) of soil microbial communities and environmental factors (pH, EC, TN). **(A)** RDA of the bacterial community; **(B)** RDA of the fungal community. The first two axes explained 72.1% and 75.79% of the total variation in community structure, respectively. IL, goji–alfalfa intercropping; ML, goji berry monoculture; N0–N3, nitrogen application rates of 0, 150, 300, and 450 kg·hm^-^², respectively. Red arrows represent environmental factors.

Redundancy analysis (RDA) was performed to identify the key soil physicochemical factors driving the compositional differentiation of microbial communities at the genus level. For bacterial communities, the first two RDA axes accounted for 42.36% and 31.96% of the total variation, respectively, with a cumulative explanatory power of 74.32% (**Figure 6A**). The distribution of intercropping (IL) and monocropping (ML) samples was clearly separated along the RDA1 axis. The ILN1 treatment was distinctly separated from other intercropping treatments (ILN0, ILN2, ILN3) in the ordination space. The dominant bacterial genera including *Luteimonas*, *Lysobacter*, and *Kocuria* were positively correlated with RDA1, while *Nitrosospira*, *Devosia*, and *Sphingomonas* were negatively correlated with RDA1. For fungal communities, the first two RDA axes explained 43.22% and 31.92% of the total variation, respectively, with a cumulative explanatory power of 75.14% (**Figure 6B**). In the intercropping system, ILN1 showed a clear separation from the other nitrogen treatments. The dominant fungal genera *Beauveria* and Dothidotthiaceae_gen_Incertae_sedis were negatively correlated with RDA1, while *Geosmithia* and *Kocuria* showed positive correlations with RDA1. Environmental fitting analysis confirmed that the community structure of both bacteria and fungi was significantly shaped by soil pH, EC, and TN. For bacterial communities, EC (r² = 0.557, *P* = 0.001) and TN (r² = 0.365, *P* = 0.010) were significant driving factors, whereas the effect of pH was not significant (*P* = 0.309). For fungal communities, pH (r² = 0.775, *P* = 0.0005) and EC (r² = 0.872, *P* = 0.0005) showed highly significant effects on community structure, and TN also exhibited a significant effect (r² = 0.283, *P* = 0.031).

### 3.3 Effects of cropping pattern and nitrogen application rate on the α-diversity of root zone Soil Microbial Communities

#### 3.3.1 Bacterial α-diversity

Cropping pattern, nitrogen application rate, and their interaction exerted highly significant effects on soil bacterial α-diversity indices (with the exception of the Simpson index) (*P* < 0.01; **Table 4**). The effects of cropping pattern on bacterial community richness varied markedly across nitrogen levels. Under the N3 and N2 treatments, the goji–alfalfa intercropping pattern exhibited higher Chao1 and Observed_features indices than the monoculture pattern, increasing by 55.22% and 46.11%, and by 2.77% and 0.20%, respectively. However, under these same nitrogen levels, the Pielou_e, Shannon, and Simpson indices were reduced in the intercropping pattern by 4.82%, 1.32%, and 1.31%, and by 5.95%, 6.21%, and 1.10%, respectively. The opposite trend of richness and evenness reflected that under the condition of medium and high nitrogen, although the intercropping mode significantly promoted the enrichment of bacterial species in the root zone, it increased the relative abundance of dominant bacteria, thereby reducing the uniformity of species distribution in the community. Under the N1 treatment, no significant differences in bacterial α-diversity indices were observed between the intercropping and monoculture patterns (*P* > 0.05). With respect to nitrogen application rate, under the goji–alfalfa intercropping pattern, the N1 treatment resulted in higher Chao1, Observed_features, Pielou_e, Shannon, and Simpson indices than the N0 treatment, with increases of 11.80%, 7.36%, 3.75%, 4.12%, and 1.01%, respectively. Compared with the N2 treatment, the corresponding increases under N1 were 13.14%, 10.99%, 5.06%, 5.70%, and 1.12%, respectively. Overall, the ILN1 treatment exhibited a 40.83% higher Chao1 index than the MLN3 treatment, indicating that this combination represented the optimal interaction for balancing bacterial richness and evenness.

**Table 4:** Soil bacterial α-diversity indices under different treatments.

Cropping pattern	Nitrogen application rate	Chao1 index	Observed_features index	Pieloue index	Shannon index	Simpson index
IL	N3	5739.460a	5301a	0.790b	9.781a	0.983a
	N2	3717.230d	3593d	0.790b	9.375ab	0.985a
	N1	4205.590c	3969c	0.830a	9.909abc	0.996a
	N0	3761.760d	3697cd	0.800b	9.517bc	0.986a
ML	N3	3697.790d	3628d	0.830a	9.912bcd	0.996a
	N2	3617.130d	3586d	0.840a	9.996cd	0.996a
	N1	5141.660b	4872b	0.840a	10.360cd	0.997a
	N0	5878.860a	5557a	0.840a	10.539d	0.997a
	Analysis of variance					
	C	**	**	**	**	ns
	N	**	**	**	**	ns
	C×N	**	**	**	**	ns

C represents the cropping pattern, N represents the nitrogen application level, and C×N represents their interaction. Different lowercase letters indicate differences among treatments within the same year. ** indicates a highly significant difference (*P* < 0.01), and ns indicates no significant difference.

#### 3.3.2 Fungal α-diversity

Nitrogen application rate exerted a significant effect on fungal community richness (*P* < 0.05), whereas cropping pattern and the interaction between cropping pattern and nitrogen application rate had no significant effects on fungal α-diversity (*P* > 0.05; **Table 5**). Across nitrogen levels, the effects of cropping pattern were dependent on the nitrogen regime. Under the N3 treatment, the goji–alfalfa intercropping pattern exhibited higher Chao1, Observed_features, Pielou_e, Shannon, and Simpson indices than the monoculture pattern, with increases of 34.23%, 34.03%, 5.73%, 10.46%, and 5.07%, respectively. Under the N2 treatment, the corresponding changes were 4.13%, 2.77%, -1.75%, -1.47%, and 1.73%, whereas under the N1 treatment, these indices decreased by 22.25%, 22.28%, 2.03%, 5.54%, and 0.00%, respectively, in the intercropping pattern relative to the monoculture pattern. With respect to nitrogen application rate, under the goji–alfalfa intercropping pattern, the N2 treatment resulted in higher Chao1, Observed_features, Pielou_e, Shannon, and Simpson indices than the N0 treatment, with increases of 12.45%, 13.80%, 34.45%, 37.03%, and 12.44%, respectively. In contrast, under the goji berry monoculture pattern, the N1 treatment exhibited higher fungal α-diversity indices than the N3 treatment, increasing Chao1, Observed_features, Pielou_e, Shannon, and Simpson indices by 29.36%, 28.50%, 20.68%, 25.30%, and 10.14%, respectively. Overall, these results indicate that although the main effect of cropping pattern on fungal α-diversity was not significant, intercropping tended to alleviate the suppressive effects of high nitrogen input on fungal richness, whereas the monoculture pattern was more conducive to maintaining fungal community evenness under low-nitrogen conditions.

**Table 5:** Soil fungal α-diversity indices under different treatments.

Cropping pattern	Nitrogen application rate		Chao1 index	Observed_features index	Pieloue index	Shannon index	Simpson index
IL	N3	1101.919a		1091a	0.517d	5.218b	0.912b
	N2	1056.509ab		1039ab	0.562c	5.629b	0.94a
	N1	825.581d		813d	0.578bc	5.591b	0.955a
	N0	939.555c		913c	0.418f	4.108d	0.836d
ML	N3	820.905d		814d	0.489e	4.724c	0.868c
	N2	1014.566b		1011b	0.572bc	5.713ab	0.924b
	N1	1061.894ab		1046ab	0.590b	5.919ab	0.956a
	N0	1053.400ab		1046ab	0.609a	6.105a	0.955a
	Analysis of variance						
	C	ns		ns	ns	ns	ns
	N	*		*	*	*	ns
	C×N	ns		ns	ns	ns	ns

C represents the cropping pattern, N represents the nitrogen application level, and C×N represents their interaction. Different lowercase letters indicate differences among treatments within the same year. * indicates a significant difference (*P* < 0.05), and ns indicates no significant difference.

### 3.4 Effects of cropping pattern and nitrogen application rate on the β-diversity of root zone soil microbial communities

#### 3.4.1 Bacterial β-diversity

**Figure 7:**
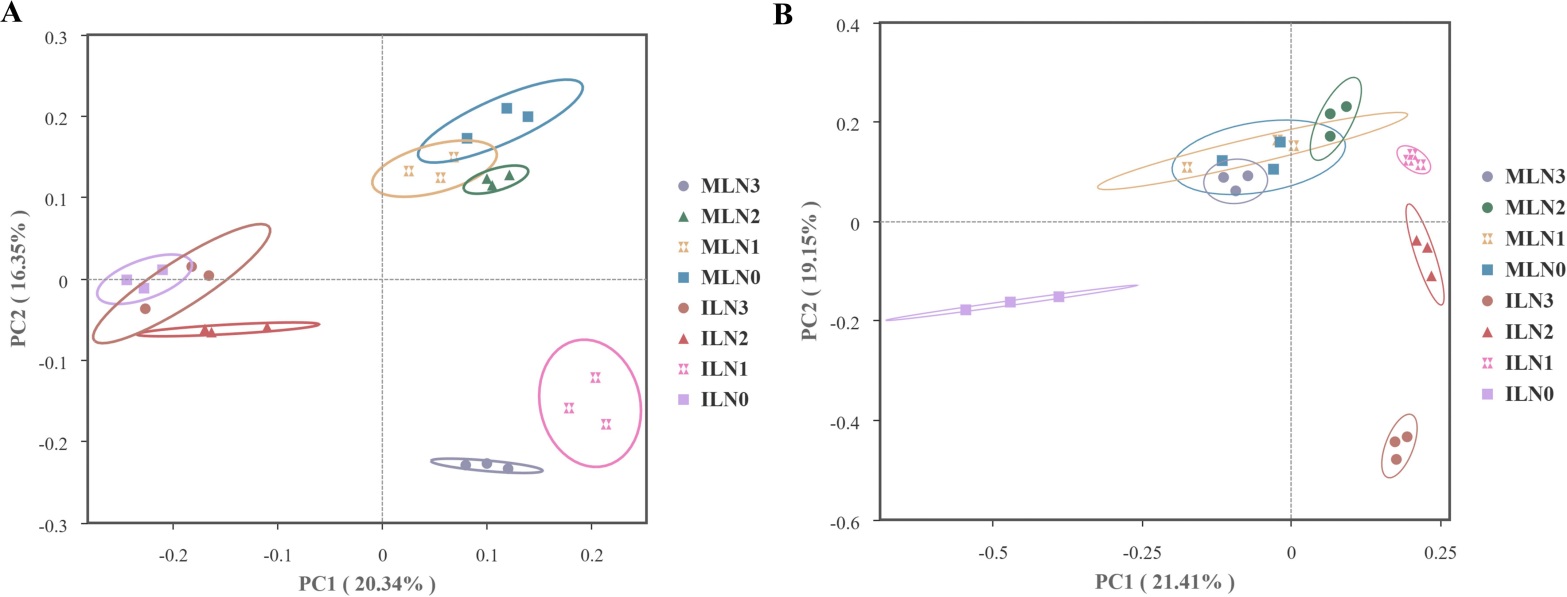
Principal Coordinate Analysis (PCoA) of microbial β-diversity under different treatments (based on Bray-Curtis distance). **(A)** Bacterial β-diversity; **(B)** Fungal β-diversity. The first two PCoA axes explained 36.69% and 40.56% of the total variation in bacterial and fungal community composition, respectively. IL, goji-alfalfa intercropping; ML, goji berry monoculture; N0–N3, nitrogen application rates of 0, 150, 300, and 450 kg·hm^-^², respectively.

Principal coordinates analysis (PCoA) was employed to evaluate the overall differences in bacterial community structure among treatments (non-metric multidimensional scaling (NMDS) analysis is provided in the **Supplementary Figure 1**). The PCoA results showed that the first two principal coordinates (PCoA1 and PCoA2) explained 20.34% and 16.35% of the total variation, respectively, accounting for 36.69% of the variation in bacterial community composition across treatments (**Figure 7A**). Along the cropping pattern gradient, samples from goji–alfalfa intercropping (IL) showed a clear separation trend from those of goji berry monocropping (ML) on both PCoA1 and PCoA2 axes. For the nitrogen application rate gradient, under intercropping, N1-treated samples were distinctly separated from N3, N2, and N0 samples; a similar pattern was observed under monocropping, where N3 samples diverged from the other nitrogen treatments. Analysis of similarities (ANOSIM) based on Bray–Curtis distances further confirmed the significance of community structure differences. (Li et al., 2018; Wang et al., 2022) The results revealed highly significant differences in bacterial community structure among the 8 different treatments (*P* < 0.01). Both cropping pattern (intercropping *vs* monocropping) and nitrogen application rate (N0 *vs* N1 *vs* N2 *vs* N3) exerted highly significant effects on bacterial community structure (*P* < 0.01).

#### 3.4.2 Fungal β-diversity

Principal coordinates analysis (PCoA) was used to assess the effects of different treatments on the overall structure of fungal communities (non-metric multidimensional scaling (NMDS) analysis is provided in the **Supplementary Figure 2**). The PCoA results showed that the first two principal coordinates (PCoA1 and PCoA2) explained 21.41% and 19.15% of the total variation, respectively, together accounting for 40.56% of the variation in fungal community composition across treatments (**Figure 7B**). In the PCoA ordination space, samples from different nitrogen application rates under the goji berry monocropping (ML) pattern were tightly clustered along the PCoA1 axis, with substantial overlap among treatments. In contrast, under the goji–alfalfa intercropping (IL) pattern, samples from different nitrogen treatments showed a clear separation trend along PCoA1 with no obvious overlap; notably, the N0 treatment formed a distinct cluster located in the negative quadrant. Combined with the quadrant distribution in the PCoA, these results indicate a pronounced differentiation trend in fungal community structure under the intercropping pattern, particularly between the N0, N1 treatments and the other nitrogen treatments. By contrast, under the monocropping pattern, samples from different nitrogen treatments exhibited considerable overlap in the PCoA space, suggesting minor differences in fungal community composition. Analysis of similarities (ANOSIM) based on Bray–Curtis distances further validated the statistical significance of these community differences (Li et al., 2023; Wang et al., 2022). The results demonstrated highly significant differences in fungal community structure among the 8 distinct treatments (*P* < 0.01). Both cropping pattern (intercropping *vs*. monocropping) and nitrogen application rate (N0 *vs*. N1 *vs*. N2 *vs*. N3) exerted highly significant effects on shaping the fungal community structure (*P* < 0.01).

### 3.5 Effects of cropping pattern and nitrogen application rate on functional gene prediction of root zone soil microorganisms of goji berry

#### 3.5.1 Bacterial functional prediction

**Figure 8:**
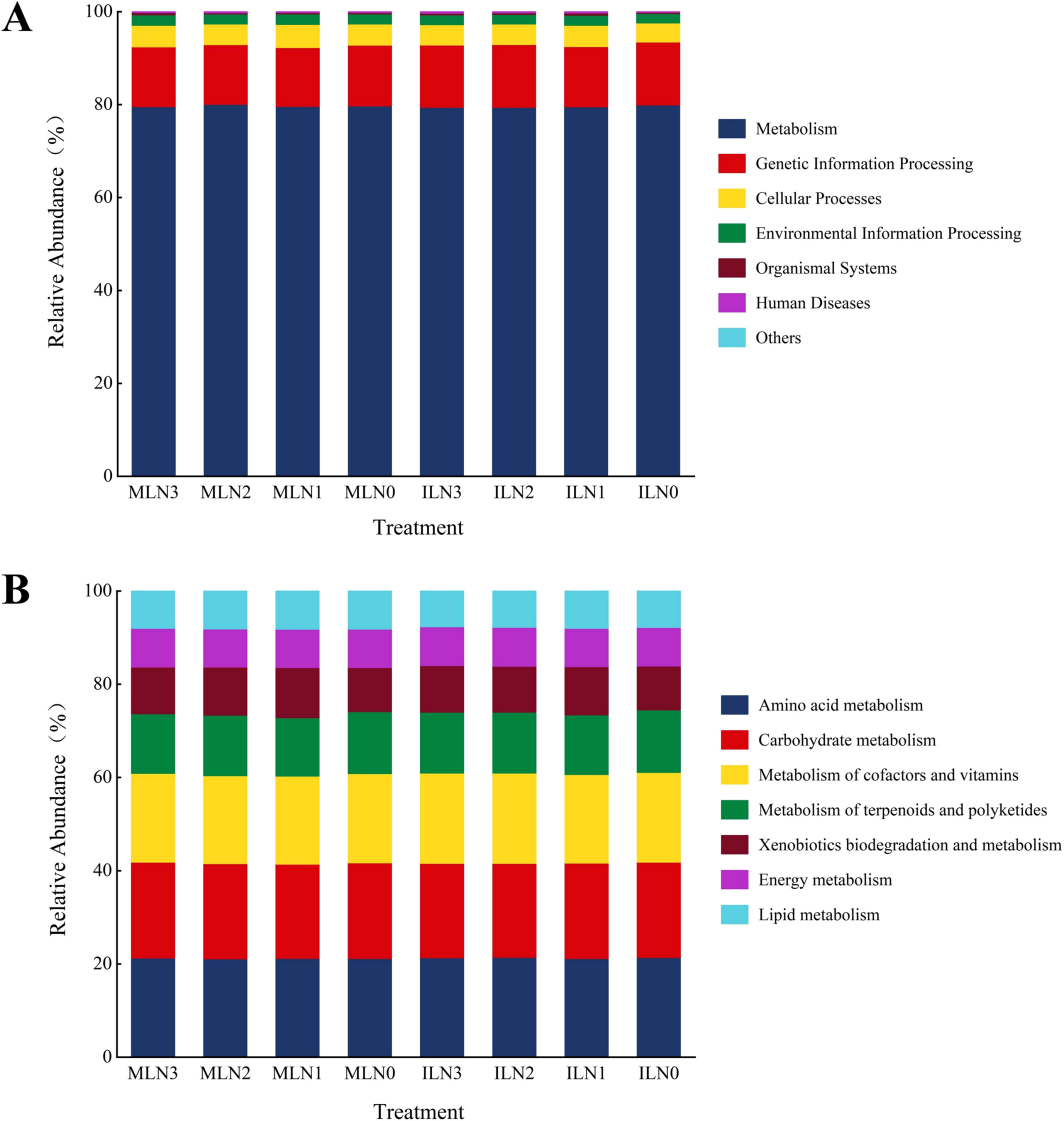
Relative abundance of predicted bacterial functions under different treatments (based on PICRUSt2 and KEGG database). **(A)** KEGG level 1 functional layer; **(B)** KEGG level 2 functional layer. IL: goji-alfalfa intercropping; ML: goji berry monoculture; N0-N3: Nitrogen application rates of 0, 150, 300, 450 kg·hm^-^², respectively.

Bacterial community functions were predicted using PICRUSt2, and the sequencing data were annotated against the KEGG database. A total of seven major categories of identifiable biological metabolic pathways were detected at the KEGG level 1 (**Figure 8A**): *Metabolism*, *Cellular Processes*, *Organismal patterns*, *Environmental Information Processing*, *Human Diseases*, *Genetic Information Processing*, and *Others.* Among these categories, *Metabolism* accounted for the largest proportion across all treatments, ranging from 79.37% to 80.02%, indicating that bacterial communities in the root zone soil of goji berry were mainly involved in metabolic activities. At KEGG level 2, eleven functional categories were identified (**Figure 8B**), including *Amino acid metabolism*, *Carbohydrate metabolism*, *Energy metabolism*, *Lipid metabolism*, *Metabolism of cofactors and vitamins*, *Metabolism of terpenoids and polyketides*, *Xenobiotics biodegradation and metabolism*, *Folding, sorting and degradation*, *Replication and repair*, *Metabolism of other amino acids*, and *Others*. Excluding the “*Others*” category, *Amino acid metabolism* exhibited the highest relative abundance across all treatments (14.16%–14.39%), representing a core pathway associated with nitrogen metabolism. This was followed by *Carbohydrate metabolism* (13.51%–13.78%), which constitutes a central pathway for energy supply, and *Metabolism of cofactors and vitamins* (12.71%–12.99%), a key pathway involved in enzyme activation and regulation of cellular metabolic processes. Notably, the ILN1 treatment (goji-alfalfa intercropping with low nitrogen) showed the highest relative abundance of functional genes related to Amino acid metabolism and nitrogen cycling among all treatments, which was consistent with the stable bacterial community structure observed in the RDA ordination and the enhanced nutrient cycling capacity suggested in the discussion.

#### 3.5.2 Fungal functional prediction

**Figure 9:**
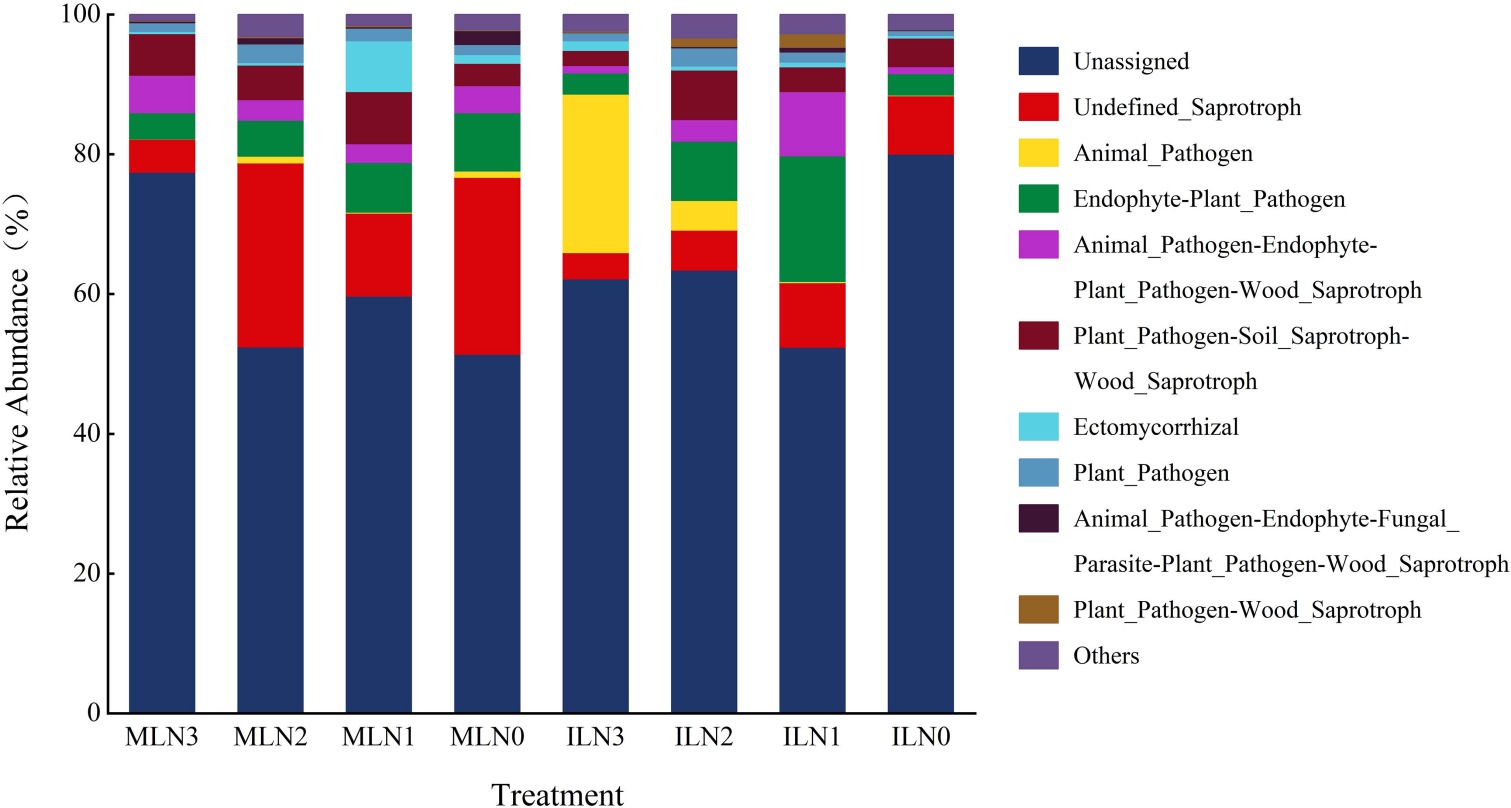
Relative abundance of predicted fungal Functions under different treatments. IL: goji-alfalfa intercropping; ML: goji berry monoculture; N0-N3: Nitrogen application rates of 0, 150, 300, 450 kg·hm^-^², respectively. Different functional guilds were classified according to the FUNGuild database annotation results.

Fungal functional guilds in the root zone soil of goji berry under different treatments were classified and predicted using FUNGuild (**Figure 9**). Statistical analysis revealed that the relative abundances of different functional fungal groups varied significantly among cropping patterns and nitrogen application levels (*P* < 0.05). The identified functional guilds included *Plant_Pathogen–Wood_Saprotroph*, *Animal_Pathogen–Endophyte–Fungal_Parasite–Plant_Pathogen–Wood_Saprotroph*, *Plant_Pathogen*, *Ectomycorrhizal*, *Plant_Pathogen–Soil_Saprotroph–Wood_Saprotroph*, *Animal_Pathogen–Soil_Saprotroph–Wood_Saprotroph*, *Endophyte–Plant_Pathogen*, *Animal_Pathogen*, *Undefined_Saprotroph*, *Unassigned*, and *Others*. Under the goji–alfalfa intercropping pattern, the N1 treatment exhibited the highest relative abundance of *Undefined_Saprotroph* (9.20%), representing an increase of 146.00% compared with the N3 treatment. Among potentially harmful guilds, *Animal_Pathogen* was enriched exclusively under the N3 treatment (22.68%) and was significantly higher than under the N2, N1, and N0 treatments. Among beneficial functional guilds, the relative abundance of *Endophyte–Plant_Pathogen* was highest under the N1 treatment (17.98%), showing an increase of 489.5% compared with the N3 treatment. Under the monocropping pattern, the N2 treatment showed the highest proportion of *Undefined_Saprotroph* (26.35%), which was 458.3% higher than that under the N3 treatment. The relative abundance of *Ectomycorrhizal* peaked under the N1 treatment (7.26%) and was significantly higher than under the other monocropping treatments. The N3 treatment exhibited the highest proportion of *Animal_Pathogen–Endophyte–Fungal_Parasite–Plant_Pathogen–Wood_Saprotroph* (5.39%), representing a 38.9% increase compared with the N0 treatment. Functional guilds associated with *Plant_Pathogen* were relatively more abundant under the N1 and N2 treatments; however, their overall proportions remained lower than those observed under the ILN1 treatment.

## 4 Discussion

### 4.1 Effects of cropping pattern and nitrogen application level on culturable microbial abundance and community composition

The present study demonstrated that cropping pattern, nitrogen application level, and their interaction exerted highly significant effects on soil culturable microbial abundance (*P* < 0.01). With respect to cropping pattern, Zhang Hongwei (Zhang, 2025). reported that a maize–soybean intercropping pattern significantly increased the abundances of soil culturable bacteria and actinomycetes, while markedly suppressing culturable fungal abundance. Similarly, He Shengran et al (He et al., 2024). observed that, under an alfalfa–sweet sorghum intercropping pattern, the root zone soil of sweet sorghum harbored significantly higher abundances of culturable bacteria and actinomycetes compared with monocropping. compared with monocropping. The similar regulatory trend observed in the goji-alfalfa intercropping system of this study stems from the fact that it, along with the maize-soybean and alfalfa-sweet sorghum intercropping systems, belongs to the category of legume-involved intercropping systems. Alfalfa supplements available nitrogen for rhizospheric bacteria and actinomycetes through symbiotic nitrogen fixation with rhizobia, and its root exudates with allelopathic effects also exert directional inhibition on fungal communities. This constitutes the common intrinsic mechanism underlying the regulation of culturable microbial groups in such intercropping systems. However, Wang Minggui et al (Wang et al., 2022). reported that both fungal and actinomycete abundances were significantly higher under a jujube–alfalfa intercropping pattern than under monocropping. Such discrepancies may be attributed to differences in functional traits among intercropped species, as well as to the heterogeneity of soil microenvironments (e.g., nutrient distribution patterns and the composition of root exudates) generated by different crop combinations. For instance, the root exudates of jujube and goji berry may exert distinct selective pressures on fungal communities, and the underlying mechanisms warrant further investigation. Our results further indicated that the effects of nitrogen application were strongly dependent on cropping pattern. Under the goji–alfalfa intercropping pattern, the responses of culturable microbes to nitrogen input were differentiated: culturable bacterial abundance was optimized at a moderate nitrogen level, culturable actinomycetes exhibited greater tolerance to low nitrogen conditions, and culturable fungal abundance was promoted under low to moderate nitrogen levels. In contrast, under the monocropping pattern, the responses of culturable microbes to nitrogen were relatively uniform, with moderate to high nitrogen levels stimulating bacterial proliferation, culturable fungal abundance increasing significantly under moderate nitrogen input, and culturable actinomycetes showing no pronounced response. Overall, these findings suggest that cropping pattern reshapes soil microbial niches by modifying root exudation profiles and nutrient competition dynamics, thereby regulating the response strategies of soil culturable microbial communities to nitrogen inputs. It should be noted that the CFU method only quantifies culturable microorganisms and has inherent limitations. However, the trends observed in CFU data were consistent with those from high-throughput sequencing (e.g., under ILN1, increased bacterial or actinomycete CFU corresponded with enrichment of beneficial genera such as *Luteimonas* and *Lysobacter*, while decreased fungal CFU aligned with reduced pathogenic phyla like *Zoopagomycota*), providing cross-validation that strengthens the reliability of our conclusions.

To further elucidate the regulatory mechanisms by which cropping pattern and nitrogen application level shape soil microbial communities, the community structures of bacteria and fungi were analyzed in detail. At the bacterial phylum level, the dominant taxa were consistent across the two cropping patterns, comprising *Pseudomonadota*, *Acidobacteriota*, *Gemmatimonadota*, *Thermomicrobiota*, *Actinomycetota*, and *Planctomycetota*, which is in agreement with the findings of Liang Siwei et al (Liang et al., 2025). The effects of nitrogen application on bacterial community structure were also contingent upon cropping pattern. Under the goji–alfalfa intercropping pattern, the relative abundances of *Pseudomonadota* and *Actinomycetota* both peaked at the N1 nitrogen level. A similar trend was observed under monocropping; however, the relative abundance of *Pseudomonadota* was generally higher under monocropping than under intercropping, whereas *Gemmatimonadota*, *Actinomycetota*, and *Thermomicrobiota* were consistently less abundant under monocropping. When interpreted in conjunction with microbial abundance data, these results suggest that the monocropping pattern likely maintained a bacterial community of relatively small overall size dominated by *Pseudomonadota*. In contrast, the intercropping pattern supported a substantially larger bacterial community. Although the proportional abundance of *Pseudomonadota* was lower under intercropping, its absolute abundance was likely still considerable, while both the relative and absolute abundances of other functional groups (e.g., *Gemmatimonadota*, *Actinomycetota*, and *Thermomicrobiota*) were markedly enhanced. This pattern indicated that the bacterial community in the intercropping system underwent a compositional shift: the relative abundance of copiotrophic groups adapted to high nutrient availability (e.g., *Pseudomonadota*) decreased, while oligotrophic and stress-tolerant groups adapted to arid, saline-alkali, and low-nutrient conditions (e.g., *Gemmatimonadota*, *Thermomicrobiota*) increased. Such compositional changes may enhance the adaptability of the bacterial community to the stressful environmental conditions of arid saline-alkali soils.

With respect to the fungal community, Ascomycota was the absolute dominant phylum, accounting for 83.75%–91.77% of the total relative abundance, and represents a key driver of ecosystem carbon and nitrogen cycling (Challacombe et al., 2019). This finding is consistent with the results reported by Chen Feng et al (Chen et al., 2025). In terms of nitrogen application, under the goji–alfalfa intercropping system, moderate to high nitrogen inputs were more conducive to increasing the relative abundance of *Ascomycota*, whereas under the monocropping system, the relative abundance of *Ascomycota* increased monotonically with increasing nitrogen application rates. Notably, the goji–alfalfa intercropping system exhibited a pronounced “fungal suppression and pathogen reduction” effect. Fungal abundance was significantly reduced under intercropping, and the relative abundance of potential pathogenic taxa (e.g., *Zoopagomycota*) was markedly lower than that under monocropping. This effect may be attributed to the combined roles of “biological barriers” and “root exudate regulation” exerted by alfalfa. Specifically, alfalfa root exudates, including flavonoids and saponins, may directly inhibit pathogenic fungi, while competition for oxygen, carbon sources, and spatial niches within the rhizosphere may further reduce the likelihood of pathogen colonization and infection of goji berry. Collectively, these processes confer an ecological effect of “indirect disease suppression” under intercropping pattern. Future studies integrating metabolomic approaches are warranted to verify and mechanistically resolve these regulatory pathways.

In summary, the goji–alfalfa intercropping pattern and the monocropping patterns shaped two distinct root zone microecological patterns and exhibited clear differentiation in their responses to nitrogen availability. By leveraging the biological nitrogen fixation capacity of alfalfa and complementary resource use, the intercropping pattern established a nitrogen-enriched, bacteria-dominated root zone characterized by more balanced functional attributes, representing a typical “bacterial-type” microecosystem. In contrast, the goji berry monocropping pattern, owing to its relatively simplified nitrogen sources and greater dependence on external nitrogen inputs, tended to develop a root zone with intensified nitrogen competition and fungal dominance, corresponding to a “fungal-type” microecosystem. These contrasting patterns highlight the ecological advantages of intercropping in maintaining root zone microecological balance and reducing potential disease risks in goji berry production patterns.

### 4.2 Effects of cropping pattern and nitrogen application rate on the diversity and environmental drivers of microbial community assembly

For bacterial communities, the α-diversity analysis in the present study showed that the N0 treatment under the goji berry monocropping pattern exhibited the highest species richness. This may be because nitrogen limitation suppresses the proliferation of all taxa to a similar extent, preventing the formation of dominant groups and thereby maximizing richness. This is consistent with the findings of Li et al (Li and Wu, 2018) who reported that low nitrogen input may be beneficial to the improvement of soil microbial diversity and species richness. However, more importantly, the ILN1 treatment achieved an optimal balance between richness and evenness, avoiding the low evenness observed under ILN3 and the insufficient richness under ILN2 and ILN0. Therefore, the bacterial community under ILN1 maintained both high richness and high evenness, indicating that the synergistic regulation of cropping pattern and nitrogen input may have achieved the optimization of community structure. This structure may imply enhanced functional coordination within the soil, laying a foundation for the provision of key ecosystem services such as efficient nutrient cycling. Notably, this optimal bacterial community structure under ILN1 was further supported by our RDA ordination results, which showed that ILN1 was distinctly separated from other intercropping treatments (ILN0, ILN2, ILN3) in the ordination space, forming a unique and stable community structure. In sharp contrast, the MLN3 treatment significantly suppressed bacterial richness, which is consistent with our environmental fitting (envfit) results. The results showed that bacterial communities are significantly driven by electrical conductivity (EC, r² = 0.557, *P* = 0.001) and total nitrogen (TN, r² = 0.365, *P* = 0.010), indicating that bacteria are highly sensitive to changes in soil salinity and nitrogen content. Excessive nitrogen input (N3) under the monocropping pattern may alter EC and TN gradients, thereby disrupting the bacterial community structure and reducing its richness. In addition, high nitrogen input may induce soil acidification or salinity stress, thereby deteriorating the microbial habitat and inhibiting the growth of nitrogen-sensitive taxa. This phenomenon is also supported by the study of Shen et al (Shen et al., 2010), who found that excessive nitrogen input inhibits bacterial diversity in cropland soils, and the inhibitory effect may be attributed to the monopolization of root zone carbon and nitrogen resources by dominant taxa, leading to the exclusion of oligotrophic bacteria due to resource scarcity. For fungal α-diversity, statistical analysis based on **Table 5** showed that the main effect of cropping pattern (C) was not significant (*P* > 0.05), and the interaction between cropping pattern and nitrogen application rate (C×N) was also not significant (*P* > 0.05). Only nitrogen application rate (N) had a significant effect on fungal α-diversity (*P* < 0.05). Although fungal richness under intercropping was numerically higher than that under monocropping at N2 and N3 levels, such differences were not statistically significant. These results indicated that fungal α-diversity was mainly regulated by nitrogen input rather than cropping pattern. Compared with bacteria, fungi showed weaker responses to cropping pattern, which may be related to their stronger tolerance to environmental changes and broader ecological niches.

β-diversity analysis, which reflects compositional differences among microbial communities under different treatments, further showed that under the goji–alfalfa intercropping pattern, the bacterial communities in the N3, N2, and N0 treatments exhibited a certain degree of similarity, indicating their ability to adapt to their respective nitrogen conditions. In contrast, the N1 treatment tended to separate from other nitrogen treatments, breaking this similarity and suggesting potential changes in community composition. This separation was further supported by RDA ordination results, in which ILN1 was distinctly separated from other intercropping treatments rather than distributed along the total nitrogen gradient, indicating that the community differentiation trend was driven by the combined effect of intercropping and moderate nitrogen input, rather than simply by total nitrogen content. This finding is consistent with the envfit results, i.e., bacterial communities are co-regulated by electrical conductivity (EC, r² = 0.557, *P* = 0.001) and total nitrogen (TN, r² = 0.365, *P* = 0.010), indicating that bacteria are highly sensitive to changes in soil salinity and nitrogen content. The moderate nitrogen input in the ILN1 treatment, combined with the salt-reducing effect of alfalfa intercropping, formed a suitable microenvironment (low salt, stable nitrogen supply), promoting the formation of a stable bacterial community structure, which further supports the superiority of the ILN1 treatment. In addition, our RDA results revealed a divergent environmental driving pattern for fungi, which were primarily structured by pH (r² = 0.775, *P* = 0.0005) and EC (r² = 0.872, *P* = 0.0005). This indicates that, unlike bacteria, the compositional differentiation (β-diversity) of fungal communities was less directly driven by nitrogen availability itself, but more by the soil chemical properties (pH and EC) that are concomitantly altered by nitrogen application. This pattern explains why nitrogen input significantly affected fungal α-diversity (**Table 5**) while not being the primary driver of fungal β-diversity. The distinct response patterns suggest that the observed shifts in the overall soil microbiome under different treatments were largely dominated by bacterial community restructuring. This is consistent with our core conclusion that intercropping with low nitrogen input fosters a stable, bacteria-dominated community structure, as the regulatory effects of this management practice (i.e., improvements in EC and TN gradients) align more closely with the ecological sensitivities of bacteria than fungi.

### 4.3 Effects of cropping pattern and nitrogen application rate on the predicted functions of soil microbial communities

The results of bacterial functional prediction indicated that bacterial metabolic potential was primarily concentrated in *Amino acid metabolism*, *Carbohydrate metabolism*, and *Metabolism of cofactors and vitamins*. Sun et al. reported that fungal community structure and function are strongly influenced by interspecific interactions (Sun et al., 2025). In the present study, fungal functional prediction revealed a clear shift in trophic strategies. Under the ILN1 treatment, fungal functional guilds exhibited mixed nutritional modes, combining the capacity to establish associations with host plants or engage in potential pathogenic interactions with saprotrophic lifestyles. This pattern represents a specialized adaptive strategy of fungi to the low-nitrogen conditions under intercropping and indirectly reflects intensified competition for nutrients between plants and fungi in intercropped, nitrogen-limited soils. Specifically, fungal functional groups tended to shift toward composite trophic modes, such as *Endophyte–plant pathogen*, as a strategy to cope with interspecific competition and uncertain resource availability. This shift suggests the presence of complex and tight interactions between fungal communities and goji berry roots, likely reflecting the coexistence of competitive and symbiotic relationships. In contrast, under the monocropping pattern, fungal community responses were more straightforward, with undefined saprotrophs becoming dominant. In the absence of nitrogen supplementation from leguminous crops, fungal communities in goji berry monocropping patterns appeared to rely more heavily on the decomposition of soil organic matter to obtain energy, while under low-nitrogen stress, they may also enhance symbiotic associations as an alternative strategy to alleviate nutrient limitation.

## 5 Conclusions and perspectives

This study demonstrates that, in arid and saline-alkali areas, goji–alfalfa intercropping combined with 150 kg·hm^-^² nitrogen application (the “intercropping + low-nitrogen” regime) can optimize the root zone microbial community, moderately increase beneficial culturable bacteria and actinomycetes, inhibit fungi and potential pathogenic guilds, promote the shift of the bacterial community toward oligotrophic taxa. Consequently, this management strategy facilitates the establishment of a highly diverse, functionally coordinated, and structurally stable “bacteria-dominated” root zone microecosystem, This model effectively constructed a root zone environment that was more unfavorable to the dominant growth of pathogens and had higher resource utilization efficiency. Overall, in arid and saline-alkali areas, goji-alfalfa intercropping with 150 kg hm^-^² of nitrogen reduction application amount not only represents the best strategy to improve the rhizosphere micro-ecological conditions, but also guides the soil process to develop in a healthier and more sustainable direction. These findings provide a solid theoretical basis for the green, efficient and sustainable development of the goji berry industry in this environmentally restricted area.

This study is based on a single sampling at the full fruit period, and the microbial community may shift with plant growth stages; thus, multi-period sampling is needed in future studies. In addition, only root zone soil was collected in this study, and differences between rhizosphere and bulk soil were not distinguished or compared. Consequently, we are unable to reveal the dynamic changes of microbial communities across the entire growing season or assess the long-term effects of interannual climate variations such as precipitation and temperature fluctuations. In addition, only soil pH, electrical conductivity and total nitrogen were determined in this study, and the analysis of key soil environmental factors such as salinity, pH, and specific nutrients that drive the differentiation of microbial communities is not deep enough due to the lack of other physicochemical indicators. Future research can do the following work:(1) To deepen the analysis of driving mechanisms, deeply correlate soil physical and chemical properties with microbial data, and accurately identify key environmental factors that drive community structure changes. (2) To carry out long-term dynamic monitoring, and to clarify the dynamic law of microbial community succession and its response to climate change by means of continuous sampling in multiple years and multiple growth periods. (3) To accurately analyze the expression activity of key microbial functional genes by combining metagenome, metatranscriptome analysis and other technologies. (4) More accurate microbial quantification methods such as qPCR can be adopted in future research to overcome the limitations of the dilution plate counting method and improve the reliability and comprehensiveness of the results. Meanwhile, we will conduct microbial co-occurrence network analysis in future studies to further verify the effects of intercropping and nitrogen application on the stability and complexity of microbial networks, and to enhance the understanding of microbial interaction mechanisms.

## Data Availability

The data presented in the study are deposited in the NCBI Sequence Read Archive (SRA) repository, BioProject accession number PRJNA1439173, BioSample accession numbers from SAMN65541639 to SAMN6554172. Further inquiries can be directed to the corresponding authors.
