# Supplementary material for: Effects of cropping patterns and nitrogen application rates on soil microbial community characteristics in goji berry root zones
Source: Front Plant Sci. 2026 Apr 2;17:1793632. doi: 10.3389/fpls.2026.1793632 (PMC13083144; doi:10.3389/fpls.2026.1793632)
Supplement: Supplementary file 1 [file DataSheet1.docx]

Supplementary Figure 1


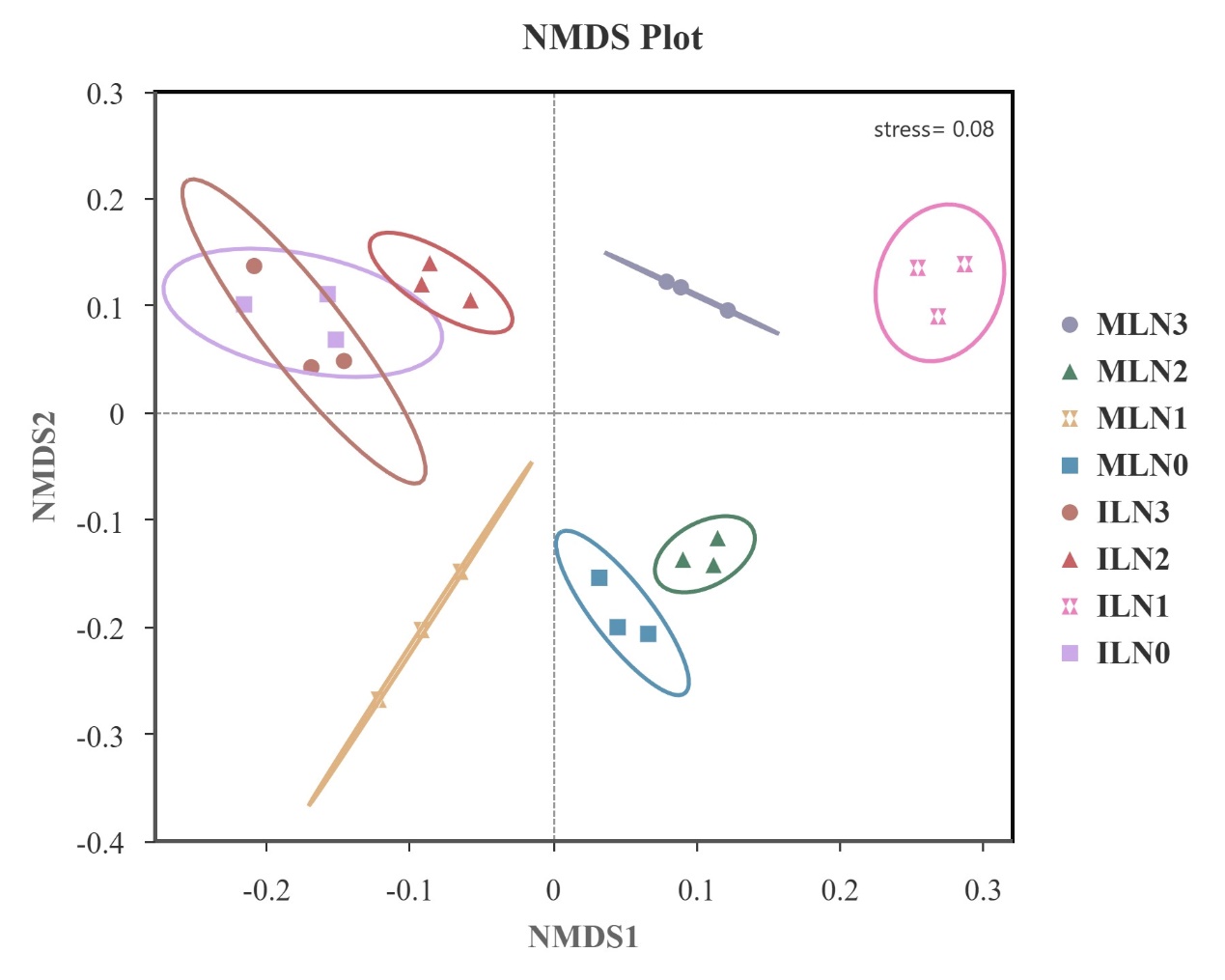


**Supplementary Figure 1** Bacterial β-diversity (NMDS plot) under different treatments (based on Bray-Curtis distance; stress=0.08). Note: IL: goji-alfalfa intercropping; ML: goji berry monoculture; N0-N3: Nitrogen application rates of 0, 150, 300, 450 kg·hm⁻², respectively. Stress value = 0.08 (Stress < 0.1, indicating a reliable NMDS ordination result).

Supplementary Figure 2


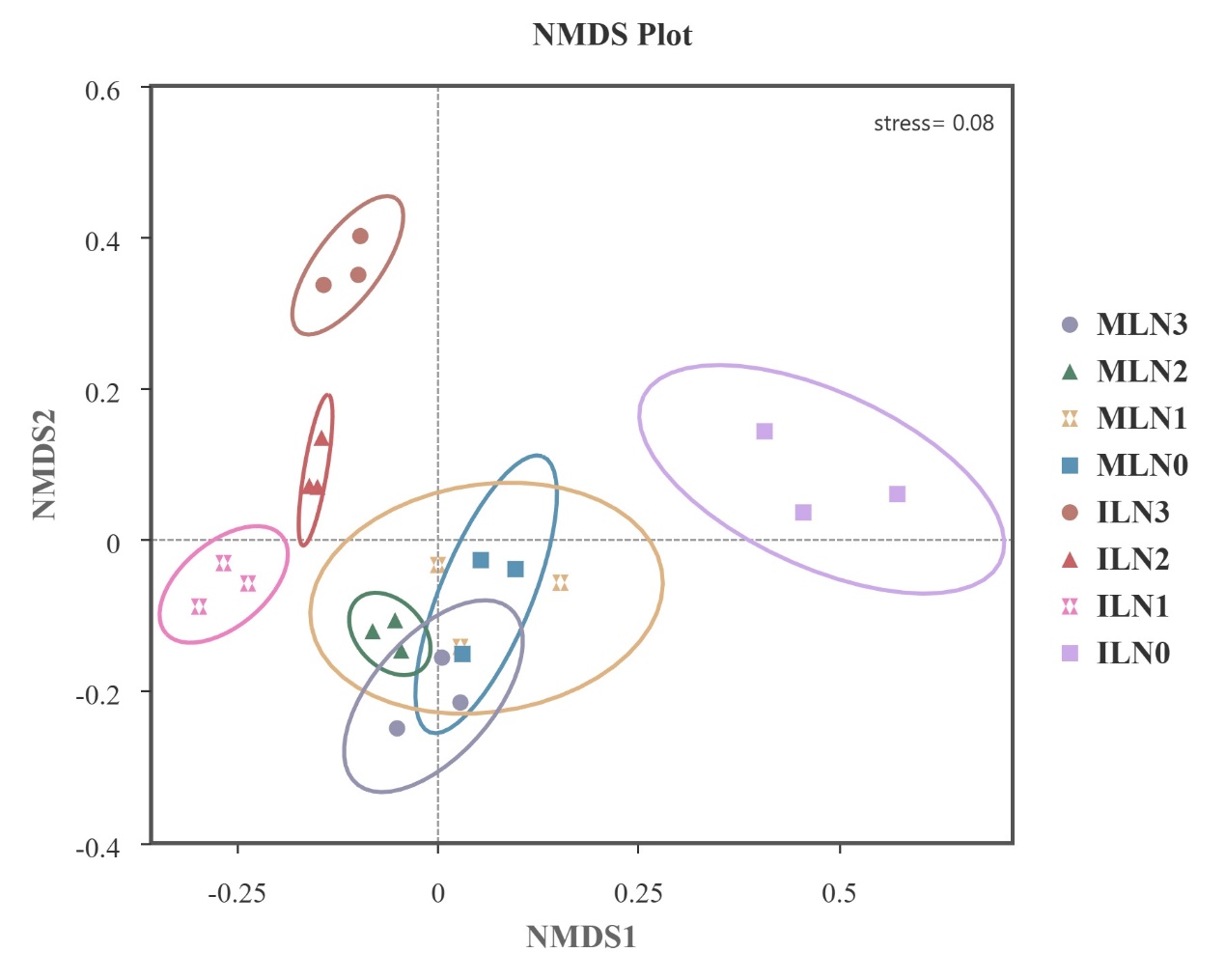


**Supplementary Figure 2** Fungal β-diversity (NMDS plot) under different treatments (based on Bray-Curtis distance; stress=0.08). Note: IL: goji-alfalfa intercropping; ML: goji berry monoculture; N0-N3: Nitrogen application rates of 0, 150, 300, 450 kg·hm⁻², respectively. Stress value = 0.08 (Stress < 0.1, indicating a reliable NMDS ordination result).
